# Epithelial-Mesenchymal-Transition-Like and TGFβ Pathways Associated with Autochthonous Inflammatory Melanoma Development in Mice

**DOI:** 10.1371/journal.pone.0049419

**Published:** 2012-11-16

**Authors:** Maria Wehbe, Saïdi M. Soudja, Amandine Mas, Lionel Chasson, Rodolphe Guinamard, Céline Powis de Tenbossche, Grégory Verdeil, Benoît Van den Eynde, Anne-Marie Schmitt-Verhulst

**Affiliations:** 1 Centre d’Immunologie de Marseille-Luminy (CIML), Aix-Marseille Université UM2, Marseille, France; 2 Institut National de la Santé et de la Recherche Médicale (INSERM), Marseille, France; 3 Centre National de la Recherche Scientifique (CNRS), Marseille, France; 4 Ludwig Institute for Cancer Research and Cellular Genetics Unit, UCL, Brussels, Belgium; Van Andel Institute, United States of America

## Abstract

We compared gene expression signatures of aggressive amelanotic (Amela) melanomas with those of slowly growing pigmented melanomas (Mela), identifying pathways potentially responsible for the aggressive Amela phenotype. Both tumors develop in mice upon conditional deletion in melanocytes of *Ink4a/Arf* tumor suppressor genes with concomitant expression of oncogene H-Ras^G12V^ and a known tumor antigen. We previously showed that only the aggressive Amela tumors were highly infiltrated by leukocytes concomitant with local and systemic inflammation. We report that Amela tumors present a pattern of de-differentiation with reduced expression of genes involved in pigmentation. This correlates with reduced and enhanced expression, respectively, of microphthalmia-associated (*Mitf*) and *Pou3f2/Brn-2* transcription factors. The reduced expression of Mitf-controlled melanocyte differentiation antigens also observed in some human cutaneous melanoma has important implications for immunotherapy protocols that generally target such antigens. Induced Amela tumors also express Epithelial-Mesenchymal-Transition (EMT)-like and TGFβ-pathway signatures. These are correlated with constitutive Smad3 signaling in Amela tumors and melanoma cell lines. Signatures of infiltrating leukocytes and some chemokines such as chemotactic cytokine ligand 2 (Ccl2) that contribute to leukocyte recruitment further characterize Amela tumors. Inhibition of the mitogen-activated protein kinase (MAPK) activation pathway in Amela tumor lines leads to reduced expression of EMT hallmark genes and inhibits both proinflammatory cytokine Ccl2 gene expression and Ccl2 production by the melanoma cells. These results indicate a link between EMT-like processes and alterations of immune functions, both being controlled by the MAPK pathway. They further suggest that targeting the MAPK pathway within tumor cells will impact tumor-intrinsic oncogenic properties as well as the nature of the tumor microenvironment.

## Introduction

Melanoma tumors arise from neural crest-derived melanocytes, cells specialized in the synthesis of melanin pigments [1]. The transition from normal melanocytes to metastatic melanomas occurs through a multistage process [2]. The acquisition of invasive behavior in cancers of epithelial origin is due in part to a phenotypic switch called epithelial-mesenchymal-transition (EMT). In this process, epithelial cells lose contacts with neighboring cells and assume migratory characteristics. EMT, described as the developmental switch undergone by cells from a polarized epithelial to a motile mesenchymal phenotype during embryonic development, has emerged as a central process of cancer progression [3]–[5]. It is characterized by decreased epithelial and increased mesenchymal markers [6]. Many EMT inducers such as TGFβ [7] have been identified, and molecular mechanisms related to the highly invasive characteristics of cancer cells have been intensively investigated [3], [6]. In particular, oncogenic Ras or activation of the MAPK pathway and TGFβ have been shown to cooperatively regulate epithelial cell plasticity and invasiveness [5], [8], [9].

We have described a mouse model of inducible melanoma based on the conditional deletion of the *Ink4a/Arf* tumor suppressor genes with concomitant expression of the *H-Ras^G12V^* oncogene and the cancer-germline gene P1A (*Trap1a*), encoding a natural mouse tumor antigen. We previously showed that two types of cutaneous tumors expressing P1A develop in induced mice: pigmented melanomas (Mela) that grow slowly and amelanotic (Amela), more aggressive tumors [10]. Both types of melanomas were deleted at the *Ink4a/Arf* locus and expressed transcripts for *H-Ras^G12V^*, albeit expression was higher in the Amela tumors [11]. The more aggressive Amela melanomas were highly infiltrated by leukocytes concomitant with local and systemic inflammation [11]. Neither tumor infiltration by leukocytes nor systemic inflammation was observed in Mela-bearing mice.

Here we compare the gene expression signatures of both tumor types, identifying pathways potentially responsible for the aggressive Amela phenotype. We show (i) a de-differentiated phenotype with reduced expression of Mitf, (ii) expression of genes akin to those defining EMT and (iii) expression of genes encoding chemokines and immuno-modulating cytokines characterizing aggressive (Amela) compared to slow progressor (Mela) melanomas. EMT-like and TGFβ-pathway signatures, correlated with constitutive Smad3 signaling, also characterized Amela tumor cells. Inhibition of the MAPK activation pathway in Amela tumor lines led to reduced Smad3 signaling, affected expression of EMT hallmark genes and inhibited proinflammatory cytokine Ccl2 gene expression and production by the melanoma cells. We conclude that in this model of autochthonous inflammatory melanoma, EMT-like processes and alterations of immune functions are linked, both being controlled by the MAPK pathway.

## Results

### Segregation of Mela, Amela and Normal Skin Gene Expression Profiles

To characterize the molecular differences between Mela and Amela tumors, we performed gene expression microarrays on 8 whole tumor samples**,** as well as on a pool of healthy mouse skins (see Methods and Fig. S1). Skin from control mice was chosen for the gene expression studies because it corresponds to the environment in which the tumors develop that may have topical characteristics in terms of stromal cells and leukocytes that are also present in the tumors.

To identify more precisely the genes differentially expressed between Mela and Amela tumors, gene expression array datasets were subjected to the microarray data analysis program Significance Analysis of Microarrays (SAM) [12]. This analysis revealed 1195 genes differentially expressed between the two types of tumors (>2 fold, p value <0.001), 813 of which were down-regulated in Amela versus Mela tumors.

Using hierarchical clustering analysis, three distinct groups were identified based on the similarity of their expression patterns. These segregated Mela from Amela tumors and both tumors from normal skin. Examples of clusters of genes are shown (i) upregulated selectively in Amela-tumors (Fig. S1A), (ii) up- and down-regulated, respectively, in Mela- and Amela-tumors (Fig. S1B), (iii) up-regulated (Fig. S1C) or (iv) down-regulated (Fig. S1D) in both Amela and Mela tumors, as compared to normal skin.

### Down-regulation of Transcripts Controlling Melanocyte Differentiation in Amela Tumors is Associated with up-regulation of *Pou3f2/Brn*2 Transcripts and Increased Aggressiveness

**Figure 1:**
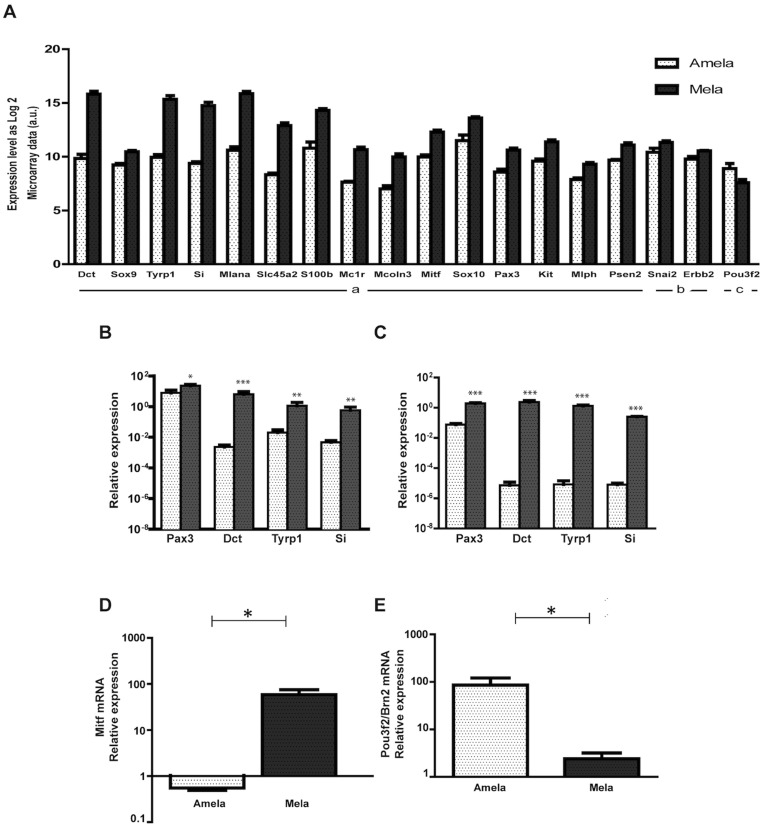
Differential expression in Amela and Mela tumors of genes involved in pigmentation, differentiation and development of melanocytes. A. Microarray data as log2 for Amela and Mela tumors are shown in arbitrary units (a.u.). For these genes, ratio of gene expression as log2 Amela/Mela is < -1 (a) or between -1 and 0 (b) with p values < 0.05; for Pou3f2 (c), ratio of gene expression as log2 Amela/Mela is > 1 with p value < 0.05. B–C. Validation of expression of four genes (from A) by QRT-PCR in Amela and Mela tumors *ex vivo* (B) and of corresponding melanoma lines cultured *in vitro* (C). D–E. Relative expression of transcripts for *Mitf* (D) and for *Brn2/Pou3f2* (E) in induced Mela and Amela tumors *ex vivo*. Amela tumors are represented by white dotted bars and Mela tumors by black dotted bars. For *ex vivo* analysis (B, D, E) values were normalized to those for skins of control mice, 6 samples of each tumor and 4 skin samples were analyzed. For *in vitro* analysis (C), 3 different cDNA preparations from two Mela and 8 Amela tumor lines were used and values were normalized to those for B16F10 cells. ***p value < 0.001; **p value < 0.01; *p value < 0.05 (see methods).

According to microarray data, transcripts controlling pigmentation, keratinization and epidermis development were down-regulated in Amela versus Mela tumors (Fig. 1A-Table S1). These include (Fig. 1A–E) genes (*Tyrp1, Si, Mlana*) encoding melanosomal proteins (Tyrosinase related protein 1/gp75, Silver/gp100, Melan-A/Mart-1), as well as genes encoding transcription factors Mitf, Pax3 and Sox10, known to be required for expression of the melanosomal proteins (for review [13]). This pattern of gene expression was verified by quantitative reverse transcriptase-polymerase chain reaction (QRT-PCR) in Amela versus Mela tumors (Fig. 1B), as well as in the corresponding tumor lines (Fig. 1C). QRT-PCR data also confirmed that the gene encoding the master transcription factor for melanocyte differentiation (*Mitf*), which is highly expressed in Mela tumors, was down-regulated in Amela tumors (Fig. 1D). In man, a correlation between MITF expression in primary melanomas and survival has been described, with de-differentiated melanomas being associated with poor prognosis [14]. Interestingly, the *Pou3f2* gene encoding a transcription factor also called Brn2 showed higher expression in Amela compared to Mela tumors (Fig. 1A, E). This is consistent with data showing *Brn2* expression up-regulation by Ras and MAPK signaling [15] and BRN2 repression of MITF expression in some human melanoma cell lines [16]. These results are in agreement with the reported correlation between the aggressiveness of human melanomas and down-regulation of expression of genes controlling melanocyte differentiation, including *MELANA, MC1R, PAX3 and c-KIT* [17], [18].

### Inflammatory Gene Expression Profile in Amela Tumors

Among the genes with up-regulated expression in Amela tumors, some characterize immune response components or chemotaxis (Tables 1- Table S2). To distinguish signatures of leukocytes infiltrating selectively Amela tumors from those intrinsic to the tumor, we compared gene expression profiles of whole Amela tumors with their *in vitro* derived cell lines (see Methods). This analysis permitted us to identify the transcriptional signatures for the Amela tumor infiltrates (genes down-regulated more than twofold in the Amela tumor lines compared to the whole Amela tumors) (Table S2). Most of these genes were characterized by a myeloid lineage expression, while some pertained to lymphocytes or stromal cells (Table S2), in agreement with our previous cellular analysis. Indeed, the Amela tumor infiltrate was found to be composed mainly of myeloid cells, a majority of which expressed the CD11b and Gr-1 markers, akin to “Myeloid Derived Suppressor Cells (MDSC)”, whereas T cells composed about 10% of the hematopoietic cell infiltrate [11].

**Table 1:** Amela tumor-intrinsic expressed genes controlling angiogenesis, invasion and cytokines.

Gene symbol	Gene name	(b)Log2 ratioAmela/Mela	(c)Log2 ratioAmela/Line
(a) *Angiogenesis*			
Vegfa	Vascular endothelial growth factor A	1.45	−1.71
Foxc2	Forkhead box C2	1.36	−0.40
Tnfaip2	Tumor necrosis factor, alpha induced protein 2	1.32	−0.91
Adamts1	a disintegrin-like and metallopeptidase (reprolysin type) with thrombospondin type 1, motif, 1	1.12	0.07
(a) *Proliferation and invasion*			
Igfbp3	Insulin-like growth factor binding protein 3	3.15	0.64
Lox	Lysyl oxidase	2.5	0.33
Tbx3	T-box 3	2.4	−0.4
Stat4	Signal transducer and activator of transcription 4	2.10	−1.62
Igfbp4	Insulin-like growth factor binding protein 4	1.70	1.64
Axl	AXL receptor tyrosine kinase	1.68	0.07
Socs3	Suppressor of cytokine signaling 3	1.31	0.08
Loxl2	Lysyl oxidase-like 2	1.2	0.17
(a) *Immune response and chemotaxis*			
Ccl2	Chemokine (C-C motif) ligand 2	2.25	−2.46
Cxcl5	Chemokine (C-X-C motif) ligand 5	2.19	0.75
IL6	Interleukin 6	1.66	0.72
Ccl5	Chemokine (C-C motif) ligand 5	1.61	−1.57
Ltbp1	Latent transforming growth factor beta binding protein 1	1.86	−0.657
Pla2g7	Phospholipase A2, group VII (platelet-activating factor acethylhydrolase, plasma)	1.55	−0.98
Chst2	Carbohydrate sulfotransferase 2	1.40	0.98
Ccl7	Chemokine (C-C motif) ligand 7	1.38	−1.03
Cxcl10	Chemokine (C-X-C motif) ligand 10	1.23	−0.48
Uaca	Uveal autoantigen with coiled-Coil domains and ankyrin repeats	1.19	0.45
F8	Coagulation factor 8	1.07	−0.11

(a) Genes characterizing angiogenesis, proliferation and invasion, immune response components or chemotaxis that show higher expression in Amela versus Mela primary tumors and are expressed at similar level in Amela primary tumors and Amela lines in culture.

(b) Ratio of gene expression as Log2 primary Amela/primary Mela > 1 with p value < 0.001;

(c) Ratio of gene expression as Log2 primary Amela/cultured Amela line < 1 or with p value n.s.).

We also identified Amela tumor-intrinsic expressed genes (Table 1). Some encode factors involved in angiogenesis such as Vegfa, or molecules involved in signaling and controlling proliferation or invasion. Interestingly, among the genes considered as signatures for leukocytes selectively infiltrating Amela tumors (Table S2), some encode receptors (*ccr1*) for chemokines encoded by genes (*ccl2, ccl5, ccl7*) expressed in Amela tumors (Table 1).

Thus, inflammation, possibly initiated by cytokines produced by Amela tumors (see later section), may be further amplified by myeloid cells recruited by tumors [11].

### Gene Set Enrichment Analysis

To identify pathways that might impart the phenotypes described for the two types of melanomas we performed gene set enrichment analyses (GSEA) using gene set 2.0 (http://www.broadinstitute.org/gsea). Specifically, an established collection of more than 100 cancer related, curated gene sets provided by the Molecular Signatures Database (MSigDB, www.broadinstitute.org/gsea/msigdb) as well as those collected from the literature, were used to interrogate the gene expression dataset in order to compare gene set enrichment in each type of melanoma.

**Figure 2:**
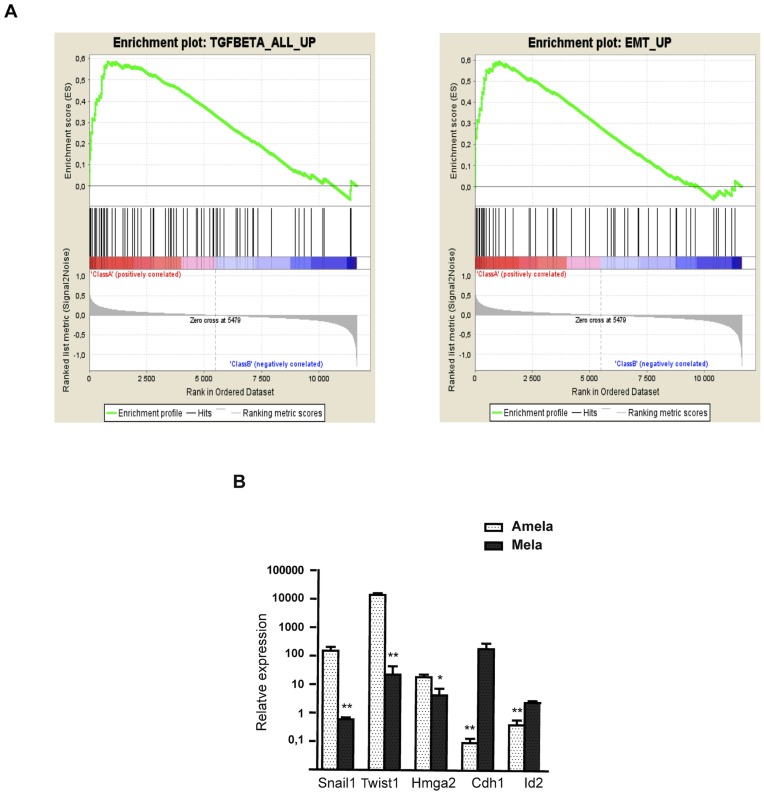
EMT and TGFβ pathway signatures in Amela melanomas. A. Representative gene set enrichment analysis **(**GSEA) plots of EMT (right graph) and TGFβ pathway (left graph) gene signatures. Each plot is divided into two sections. The first section (class A) shows results for gene sets that have a positive enrichment score (gene sets that show enrichment at the top of the ranked list here associated with Amela samples). The second section (class B) shows the results for gene sets that have a negative enrichment score (gene sets that show enrichment at the bottom of the ranked list here associated with Mela samples). Data provided as in Fig. S2. **B.** QRT-PCR analysis for expression of transcripts encoding mesenchymal and epithelial markers in Amela (white dotted bars) and Mela (black dotted bars) tumors. Values were normalized to those for skin from control mice. Data are represented as mean ± s.e.m of three independent experiments in which 7 samples of Amela and 7 samples of Mela tumors were analyzed. **p value < 0.01; *p value < 0.05.

We found 40 gene signatures specifically enriched in either the Amela tumors or in the Mela tumors (p < 0.05) (not shown). Notably, a significant number of the gene sets enriched in the Amela versus the Mela tumors were classifiers for either EMT or pathways known to induce EMT, such as TGFβ pathways (Fig. 2A-Fig. S2).

### Aggressive Amela Tumors are Associated with a Signature Akin to EMT

In agreement with the GSEA analysis, among the genes distinguishing Amela from Mela tumors and qualifying as intrinsically tumor-expressed, we identified genes encoding proteins associated with melanoma progression, akin to those characterizing EMT (Table 2). These include transcriptional regulators Hmga2, Twist1 and Snail1, whose expression is up-regulated, and Id2, whose expression is down-regulated. Changes in adhesion molecule transcripts (down-regulation of E-cadherin/*Cdh1*, up-regulation of N-cadherin/*Cdh2* and integrin alpha 5/*Itgav* transcripts) also characterize EMT.

**Table 2:** Genes up-regulated or down-regulated in Amela tumors involved in EMT.

Gene symbol	Gene name	(b)Log2 ratio Amela/Mela	(c)Log2 ratio Amela/Lines
(a)Cell adhesion/ECM matrix/Cytoskeleton			
Itgbl1	Integrin beta like 1	2.18	0.20
Lamb1–1	Laminin beta 1, subunit 1	1.47	−0.76
Sdc2	Syndecan-1	1.36	0.36
Cdh2	N-cadherin	1.25	−0.33
Col4a1	Collagen, type IV, alpha 1	0.99*	0.87
Col11a1	Collagen, type XI, alpha 1	0.81*	−0.54
Itgav	Integrin alpha 5	0.62*	−0.30
Col6a1	Collagen, type VI, alpha 1	0.57*	0.96
Itga3	Integrin alpha 3	0.46*	−1.90
(a)Proteases . Protease Iinhibitors			
Mmp10	Metallopeptidase 10	2.67	1.26
Pcolce	Procollagen C-endopeptidase enhancer protein	1.03	−0.93
Mmp19	Metallopeptidase 19	0.92*	0.20
Serpinb9	Serine peptidase inhibitor, Clade B, member9	0.72*	0.75
(a)PDGF/PDGF receptor pathway			
Ccl2	Chemokine CC motif ligand 2	2.23	−2.46
Pdgfc	Platelet dervied growth factor, C polypeptide	1.59	−0.31
Cmkor1	Chemokine CXC motif receptor 7 (CXCR7)	1.46	−0.52
Pdgfa	Platelet dervied growth factor, alpha	0.67	0.39
Pdgfd	Platelet dervied growth factor, D polypeptide	0.47	0.26
(a)Hypoxia			
Upp1	Uridine phosphorylase	1.74	−0.75
Il11	Interleukin 11	1.69	−0.97
Hif1a	hypoxia inducible factor 1, alpha subunit	0.59*	−1.46
(a)Transcription factors			
Hmga2	high mobility group alpha 2	3.71	−1.05
Twist1	Twist homolog 1 (Drosophila)	2.07	−0.78
Foxc2	Forkhead box c2	1.36	−0.40
Snai1	Snail homolog 1 (Drosophila)/Snail1	1.16	−0.07
(d) Down-regulated in Amela			
Cdh1	E-Cadherin	−3.62	2.28
Id2	Inhibitor of DNA binding 2	−0.83*	0.6

(a) Genes known to be involved in EMT that show higher expression in Amela versus Mela primary tumors and are expressed at similar level in Amela primary tumors and Amela lines in culture.

(b) Ratio of gene expression as log2 primary Amela/primary Mela > 1 with p values < 0.05;

* Ratio of gene expression as log2 primary Amela/primary Mela between 0 and 1 with p values < 0.05;

(c) Ratio of gene expression as Log2 (primary Amela-/cultured Amela line) < 1 and/or with p value > 0.05 (ns). Log2 (primary Amela /cultured Amela line) < - 1 corresponds to higher expression in cultured Amela line than in primary Amela.

(d) Genes known to be involved in EMT that show lower expression in primary Amela versus Mela tumors.

We next compared the mRNA level of different epithelial and mesenchymal markers on both types of tumors by QRT-PCR. As shown in Fig. 2B, the Amela tumors expressed higher levels of transcripts for mesenchymal markers (Snail1, Twist1 and Hmga2) than Mela tumors and a lower level of E-cadherin/*Cdh1* and *Id2* transcripts. Given recent reports implicating Snail1 in both inflammation and EMT-like processes in the skin [19] or in melanoma cells [20], we analyzed its expression by immunohistology on Amela tumors (Fig. S3). It should be noted that staining with the commercially available anti-Snail1 antibody revealed mostly cytoplasmic Snail1, although Snail1’s function as a repressor of transcription is dependent on its nuclear localization [21]. Snail1 shuttles between the cytoplasm and the nucleus in a regulated manner that is not fully understood. Post-translational modifications including phosphorylation on serine and threonine residues, ubiquitination and lysine oxidation affect Snail1 protein stability, subcellular localization and activity [21]–[24]. Thus, although Snail1 appears to be expressed in Amela tumors, further biochemical analyses would be required to analyze post-translational modifications of the protein and their influence on its localization and function.

### Genes Involved in the TGFβ Pathway in Amela Lines

Since the GSEA analysis showed a significant enrichment of genes involved in TGFβ pathways, we identified the genes upregulated in the Amela tumors known to take part in the TGFβ pathways based on the literature (Table 3). In particular, transcripts encoding components of the TGFβ pathway (Ltbp1, Igfbp3) are upregulated in Amela tumors, although no active TGFβ appeared to be secreted (see next section).

**Table 3:** Representative TGFβ Related Genes expressed in Amela tumors and cultured cell lines.

TGFβ related genes				
(a)Gene symbol	Gene name	(b)log2 ratioAmela/Mela	(c)log2 ratioAmela /Lines	(d)References
Igfbp3	Insulin-like growth factor binding protein 3	3.15	0.64	[1]
Cspg2	Versican	2.55	0.04	[2]–[4]
Itgbl1	Integrin beta-like 1	2.18	0.20	[4]
Cald1	Caldesmon 1	1.93	−0.08	[5]–[7]
Ltbp1	Latent transforming growth factor beta binding protein 1	1.78	−0.66	[8]
Nid1	Nidogen	1.72	0.66	[9]
Igfbp4	Insulin-like growth factor binding protein 4	1.60	1.64	[10]
Lamb1–1	Laminin B1 subunit 1	1.47	−0.76	[4], [7], [11]–[12]
Cmkor1	Chemokine CXC motif receptor 7 (RDC1, CXCR7)	1.46	−0.52	[2], [6], [12]–[14]
Fhl2	Four and a half LIM domains 2	1.43	−0.40	[2], [6]–[7], [12]–[13]
Lrp1	Low density lipoprotein receptor-related protein 1	1.40	0.88	[15]
Plat	Plasminogen activator, tissue	1.40	0.99	[16]
Bmp1	Bone morphogenetic protein 1	1.30	0.54	[6]–[7], [12]
Smad3	MAD homolog 3 (Drosophila)	1.19	−0.23	[17]
Slc29a1	Solute carrier family 29 (nucleoside transporters), member 1	1.18	1.00	[5], [7], [12]
Cdh6	Cadherin 6	1.18	0.28	
Rdh10	Retinol dehydrogenase 10 (all-trans)	1.11	0.29	[6]–[7], [13]
Col4a1	Collagen type IV, alpha 1	0.99*	0.87	[7], [11]–[13]
Wnt2	Wingless-related MMTV integration site 2	0.97*	0.48	[18]
Tgfbr2	Transforming growth factor receptor 2	0.96*	0.01	[19]
Marcksl1	MARCKS-like 1	0.94*	0.88	[20]
Rhoq	Ras homolog gene family, member Q	0.90*	0.37	[20]
Ext1	Extososes multiple 1	0.85*	0.43	[2], [6]–[7]
Ier3	Immediate early response 3	0.84*	−0.43	[5], [7], [12]–[13]
Tgfb3	Transforming growth factor 3	0.82*	0.23	[19]
Pdgfa	Platelet derived growth factor, alpha	0.67*	0.39	[2], [5]–[7], [21]
Col6a1	Collagen type VI, alpha 1	0.57*	0.96	[4], [7], [22]
Itga3	Integrin alpha 3	0.46*	−1.90	[4], [7], [23]
Cd44	CD44 antigen	0.41*	−1.47	[24]
Skil	SKI-like	0.40*	0.49	[2], [7], [13]
Igf2r	Insulin-like growth factor 2 receptor	0.38*	0.10	
Tgfbr3	Transforming growth factor receptor 3	0.20*	−0.23	[19]

(a) Genes known to be TGFβ responsive that show higher expression in Amela versus Mela primary tumors and are expressed at similar level in Amela primary tumors and in Amela lines in culture.

(b) Ratio of gene expression as log2 primary Amela/primary Mela > 1 with p values < 0.05;

* Ratio of gene expression as log2 primary Amela/primary Mela between 0 and 1 with p values < 0.05;

(c) Ratio of gene expression as Log2 (primary Amela/cultured Amela line) < 1 and/or with p value > 0.05 (ns). Log2 (primary Amela/cultured Amela line) < - 1 corresponds to higher expression in cultured Amela lines than in primary tumor.

(d) Numbered references can be found in Text S1.

**Figure 3:**
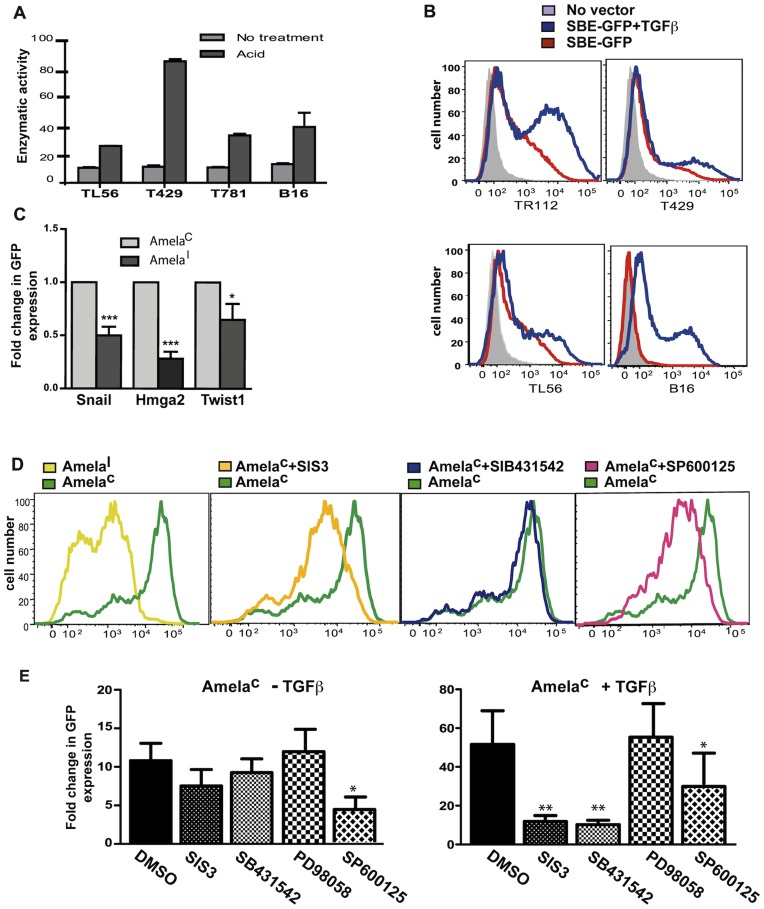
Analysis of the TGFβ pathway in melanoma lines and tumors. A. Supernatants from Amela cell lines incubated in serum-free DMEM were either acid treated (acid) or not (no treatment) and were tested for TGFβ content using reporter line MFBF11 (see Methods). Bars represent means ± s.d. of triplicate wells in one representative experiment. Serum-free DMEM (no TGFβ) was used for baseline measurement. B. Flow cytometry analysis of GFP expression in SBE-GFP-transduced Amela and B16F10 cell lines preincubated in serum-free DMEM without addition (red lines) or in the presence of TGFβ (blue lines). Non-transduced Amela cell lines were used as control (gray filled). C. Comparasion of the expression of 3 genes associated with EMT by Amela^C^ and Amela^I^ cell lines by QRT-PCR (see text). Results are represented as fold change in relative expression where the value for Amela^C^ is set to 1 for each gene. Data are represented as mean ± s.e.m of two independent experiments in which 4 different lines of each type were tested. D-E. Flow cytometry analysis of GFP expression in SBE-GFP-transduced Amela^C^ cell lines as in (B). The effect of various inhibitors was assessed on Amela^C^ cells during the incubation in serum-free DMEM without (-) or with addition (+) of TGFβ. GFP expression in one Amela^C^ line before and after treatment with various inhibitors in the absence of TGFβ is shown in (D). In (E)results show fold change of mean GFP fluorescence intensity in Amela^C^ without (panel at left) or with (panel at right) addition of TGFβ. The value for Amela^I^ in the absence of TGFβ is set to 1. Data are represented as mean ± s.e.m of four independent experiments. ***p value < 0.001; **p value < 0.01; *p value < 0.05.

TGFβ acts as tumor suppressor in early cancer. In later stages of cancer, however, TGFβ can promote tumors toward invasive and metastatic phenotypes [25], [26]. TGFβ binding to its receptor induces the formation of a heteromericcomplex composed of TGFβ receptor type II (TβRII) and I (TβRI). The latter transmits the signal through phosphorylation of receptor-regulated Smads such as Smad2 and Smad3. Both of those Smads form heteromeric complexes with Smad4 and are then translocated into the nucleus, where they regulate expression of target genes both by direct DNA binding and through interaction with other transcription factors, coactivators, and corepressors.

We used computational methods to test the hypothesis that some of the genes within the Amela up-regulated cluster are coordinately regulated by the Smad3 transcription factor. Using the Clover program, we found a significant statistical enrichment for conserved Smad binding sites in the promoters of genes highly expressed in Amela tumors as compared to background sequences. Analysis of promoter sequences identified many genes with multiple, conserved Smad binding sites (Table S3).

### Evidence for Constitutive Activation of the TGFβ Pathway in Amela cell lines and tumors

To measure TGFβ bioactivity from Amela cell culture supernatants with high sensitivity we used the SBE-SEAP reporter line MFBF11 [27] (see methods). Samples that were not activated with acid did not induce reporter activity. After activation with acid, all samples were able to strongly induce reporter activity, showing that Amela tumor cells express the latent form of TGFβ (Fig. 3A).

We transduced Amela as well as B16F10 melanoma cell lines with the lentivirus-based Smad3-reporter vector expressing green fluorescent protein (SBE-GFP) [28]. Surprisingly, even in the absence of TGFβ, we detected GFP-fluorescent Amela tumor cells, but no fluorescent B16F10 cells (Fig. 3B), although all cell populations responded to TGFβ induction. A heterogeneous constitutive GFP expression was observed in all tested Amela lines. Based on GFP expression in these cells in the presence or absence of added TGFβ, Amela cells were sorted by FACS. Amela cells expressing GFP constitutively are called Amela^C^ and Amela cells expressing GFP after TGFβ-induction are called Amela^I^. QRT-PCR analysis showed higher expression of 3 selected Smad3-regulated genes in Amela^C^ compared to Amela^I^ cells (Fig. 3C).

To explain the constitutive activation of Smad3 in Amela^C^ cells, we wondered if this population expressed active TGFβ, which was not detected when analyzing supernatants from the whole population. To test this hypothesis, supernatants of Amela^C^ and Amela^I^ cells were tested in the SBE-GFP-reporter assay. Only acid-treated supernatants from both cell types induced reporter activity, showing that the basal activation of Smad3 in Amela^C^ cells did not result from autocrine TGFβ (Fig. 3, Fig. S4A).

### Involvement of Ras Signaling Pathways in Smad Activation in Amela Tumors

Smads are modular proteins with conserved Mad Homology 1 (MH1), intermediate linker and MH2 domains. TβRI phosphorylates the COOH-terminal serine residues of Smads (pSmad3C). The linker domain may be phosphorylated (pSmad3L) by Ras-dependent kinases such as extracellular signal-regulated kinase (ERK) and c-Jun NH2-terminal kinase (JNK) [29]. We investigated the role of the Ras signaling pathways in Smad3 constitutive activity in Amela^C^ cells (Fig. 3D). Constitutive expression of GFP in the Amela^C^ cells was significantly inhibited only by JNK inhibitor, SP600125 (Fig. 3D–E). It was not inhibited by either SIS3, a specific inhibitor of TGFβ-induced Smad3 phosphorylation, or by SB431542, an inhibitor of TβRI kinase activity (Fig. 3D–E). In contrast, both SIS3 and SB431542 were efficient at inhibiting TGFβ-induced GFP expression in Amela^C^ lines. This result suggests that Ras signaling may be involved in inducing Smad3 activity in Amela^C^ cells via activation of JNK.

### EMT in Amela Tumor Cells is Controlled by MAPK Signaling Pathways

**Figure 4:**
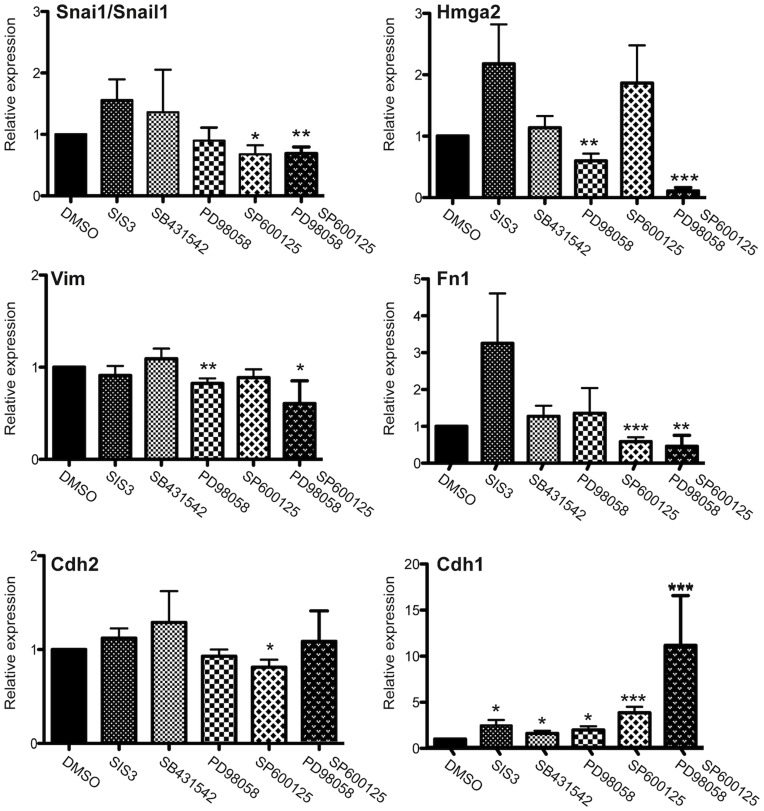
Effect of MAPK pathway inhibitors on the expression of EMT hallmark genes in Amela tumor lines. QRT-PCR analysis for expression of 6 transcripts encoding mesenchymal and epithelial markers in Amela tumor cells in the absence (value set to 1) or presence of inhibitors, as indicated. Data are represented as mean ± s.e.m of three independent experiments in which 5 samples were analyzed. ***p value < 0.001; **p value < 0.01; *p value < 0.05.

If the active Ras/MAPK pathway is responsible for activation of the transcriptional acivity of Smad3 leading to expression of an EMT-like transcriptional program, it was expected that inhibition of that pathway would lead to down-regulation of the expression of genes characterizing EMT. To test this possibility, we analyzed the effects of JNK and ERK inhibitors on the expression of various genes by QRT-PCR (Fig. 4A). The JNK inhibitor (SP600125) led to downregulation of *Snai1/Snail1, Fn1, Cdh2* and upregulation of *Cdh1* transcripts. *Hmga2* and *Vim* transcripts were decreased in the presence of the ERK (PD 98059), but not the JNK inhibitor. These data are concordant with previous results in other tumor types for Hmga2 [30]. When combining JNK and ERK inhibitors, however, a further inhibition of *Hmga2* transcripts was observed.

### Amela Tumors Express High Levels of Phosphor-Smad3L and Phosphor-JNK in vivo

**Figure 5:**
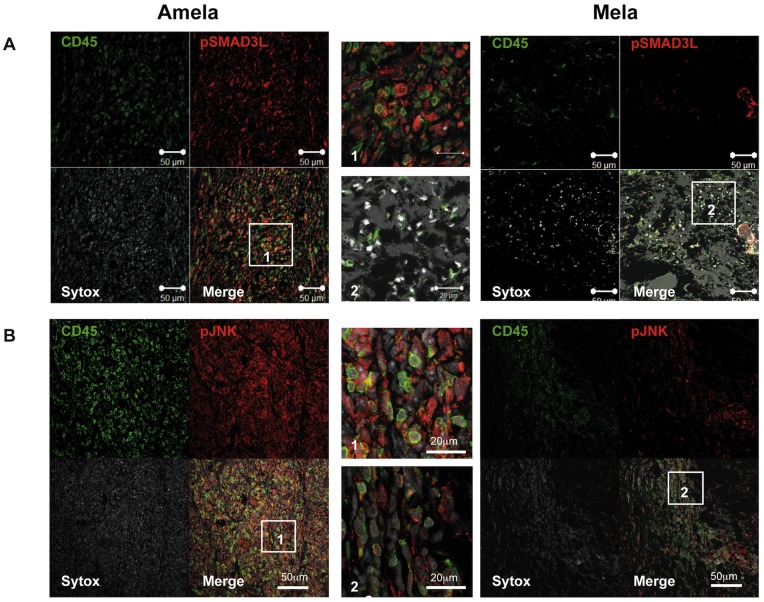
Analysis of phosphorylated Smad3 and JNK in melanoma tumors. Sections of Amela and Mela tumors were analyzed by immunohistology after staining with anti-CD45 mAb (green) and with anti-Phospho-Smad3L (pSmad3L) (A) or with anti-Phospho-JNK (pJNK) (B) (red). Sytox blue stains nuclei (white). Bars correspond to 50 µm in the 4 quadrant-figures and to 20 µm in the magnifications of the highlighted fields (labeled 1 and 2). In A, the brightfield image is superimposed on the “merge” image. Images are representative of 3 tumors of each type. Immunofluorescence was quantified using NIH ImageJ software for the determination of relative densities of expression of given markers within fixed section areas (fraction area). Values were, respectively (A) 1.95 ± 0.73 for Mela and 13.78 ± 1.55 for Amela tumor Phospho-Smad3L staining (unpaired t test p < 0.0001) and (B) 5.39 ± 1.05 for Mela and 26.36 ± 3.26 for Amela Phospho-JNK staining (unpaired t test p < 0.0005).

Given the enrichment in genes involved in the TGFβ pathway detected in the Amela tumors, we tested whether pSmad3L could be detected by immunohistology on tumor sections (Fig. 5A). Antibody to CD45 was used to identify tumor-infiltrating leukocytes together with anti-pSmad3L antibody. In the Amela tumors a high frequency of tumor cells were labeled with anti-pSmad3L antibody, whereas most infiltrating leukocytes were not labeled. In Mela tumors, few infiltrating leukocytes were detected and most tumor cells were negative or very weakly stained with anti-pSmad3L antibodies. When analyzing expression of phosphorylated JNK (pJNK), we similarly observed strong staining in Amela tumors and very weak staining in the Mela tumors (Fig. 5B). These data show a correlation between JNK activation revealed by pJNK and Smad3 phosphorylation in its linker domain, and suggest that Smad3 is constitutively active in the Amela tumors as it is in the corresponding cell lines.

### Proinflammatory Ccl2 Cytokine Production in Amela Tumor Cells is Controlled by MAPK Signaling Pathways

**Figure 6:**
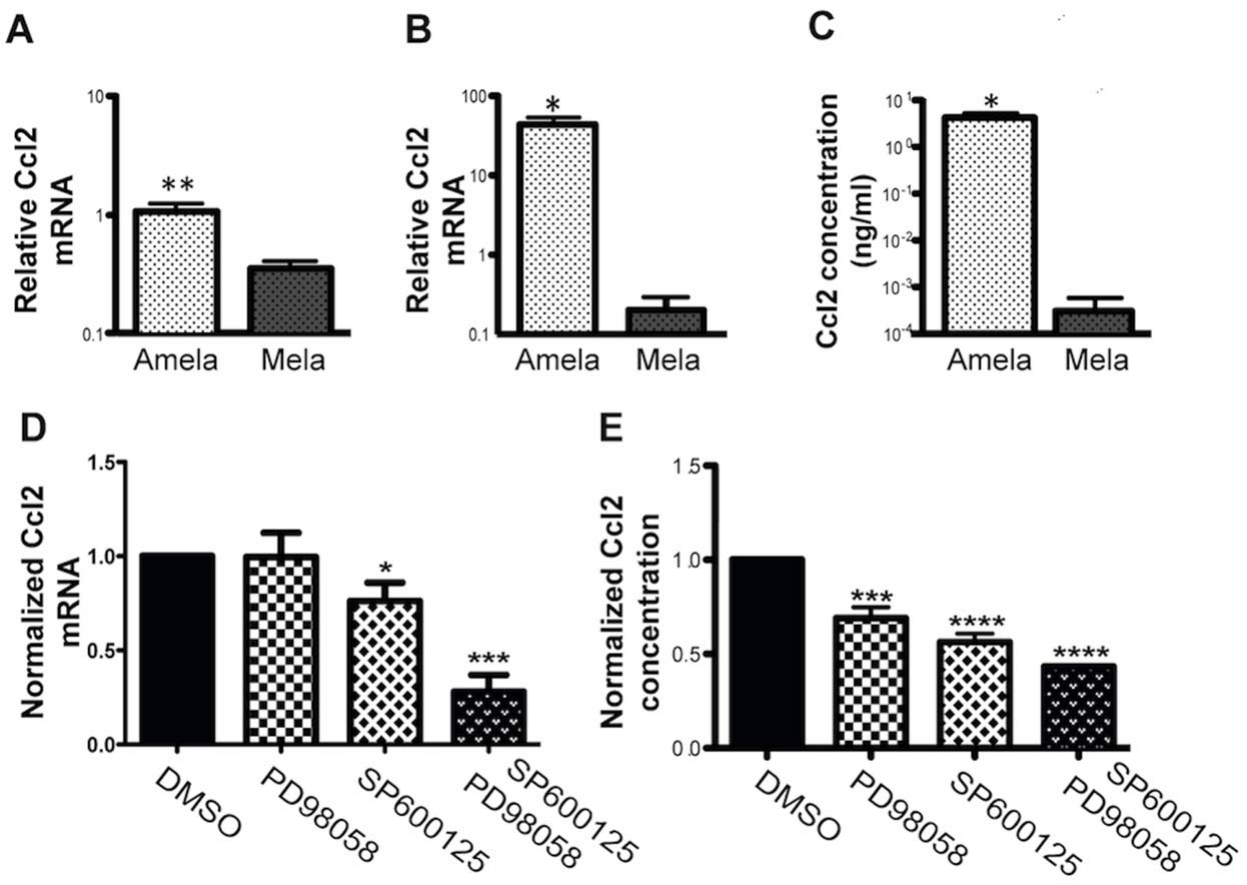
Specific expression of Ccl2 in Amela tumors is controlled by Ras signalling pathways. (A-B) QRT-PCR analysis of Ccl2 transcript expression in induced Mela and Amela primary tumors (A) and in the corresponding cell lines in vitro (B). (C) Concentration of secreted Ccl2 in the supernatant of 24 h cultures of Amela and Mela cell lines as measured by ELISA. (D) Expression of Ccl2 transcript by QRT-PCR and (E) concentration of secreted protein by ELISA, in the absence (value set to 1) or presence of inhibitors in 24 h cultures, as indicated. (A–C) Amela tumors are represented by white dotted bars and Mela tumors by black dotted bars. For primary tumor analysis (A) 6 samples of each tumor were analyzed. For *in vitro* analysis (B), values were normalized to those for B16F10 cells. In (C), supernatants from 24 h cell cultures were tested and values are expressed as ng/ml. In D–E, culture conditions were as described in Methods. Experiments involved 10 Amela lines in (D) and 4 in (E). ****p value < 0.0001; ***p value < 0.001; **p value < 0.01; *p value < 0.05.

We further analyzed the control of production of the proinflammatory cytokine Ccl2 and confirmed by QRT-PCR data the differential expression of its transcript between Amela and Mela tumors (Fig. 6A), as well as by cultured Amela and Mela tumor cell lines (Fig. 6B). Cytokine production was readily detected in 24 h supernantants of Amela, but not of Mela, nor of B16.F10 lines (Fig. 6C). We next addressed whether production of the proinflammatory cytokine was under the control of the MAPK pathway by testing the effect of the inhibitors on Ccl2 gene expression (Fig. 6D) and cytokine production (Fig. 6E) by the Amela tumor cells. Data indicate a significant inhibition of Ccl2 transcripts and cytokine production when cells were incubated in the presence of the JNK inhibitor or the ERK and JNK inhibitors (Fig. 6).

These resuts suggest that oncogenic Ras expression coordinately controls expression of an EMT and a proinflammatory gene expression program via activation of the MAPK pathway.

## Discussion

We sought to understand the molecular bases of tumor cell heterogeneity in the autochthonous TiRP mouse melanoma model. To do this, we compared gene expression profiles of the slowly progressing pigmented Mela and fast growing amelanotic Amela tumors. These tumors arise as a result of Cre-mediated deletion of the *Ink4a/Arf* genes in melanocytes together with expression of H-ras^G12V^ and P1A encoding a mouse tumor antigen. These events were observed in both types of tumors although higher levels of H-ras^G12V^ and P1A transcripts were detected in Amela tumors [10], [11]. A threshold level of H-ras^G12V^ may be required for oncogenic H-ras to signal to any of the several downstream pathways, which it can activate. These include the MAPK pathways involving notably ERK and JNK, as well as phosphoinositide-3-kinase/Akt and NF-κB pathways [31], [32]. Combined with absence of p16/Arf tumor suppressors, the level of H-ras^G12V^ may thus control key factors determining differentiation (Brn2, Mitf), EMT (JNK, Smad3, TGFβ signaling) and Ccl2 production (MAPK, JNK) during melanoma development as discussed hereafter.

Mice developing Amela melanomas presented a systemic Th2-profile of cytokines [11] associated with high expression of Vegfa by the tumors (Table 1), a situation analogous to that found in patients with metastatic melanoma [33]. *Il6* was also up-regulated selectively in the Amela melanomas (Table 1), in agreement with high level detection of Il-6 in Amela tumor supernatant and serum of Amela-bearing mice [11] and with previous reports on its induction by oncogenic Ras in different cell types [34], including melanomas in a distinct model of H-ras^G12V^-induced melanoma in mice [35].

Other cytokine genes expressed selectively by Amela tumors include those encoding Ccl2, Ccl5, and Ccl7 (Table 1), all 3 of which can contribute to the recruitment of Ccr1-expressing myeloid leukocytes (Table S2). This pattern of cytokine gene expression is thus consistent with the identified immune cell gene expression signature in the Amela tumors (Table S2) and myeloid cell infiltrate detected in the Amela tumors [11]. The latter may in turn amplify inflammation in the tumor microenvironment [11].

Tumor expression of the proinflammatory cytokine Ccl2, also called monocyte chemoattractant protein 1 (MCP1), has been associated with macrophage migration to the tumor and promotion of tumor growth in transplanted tumor models [36] and human melanoma xenografts [37]. More recently, Ccl2 has also been implicated in recruitment of MDSC to transplanted tumors [38] and to an autochthonous glioma [39] in mice. These data are in line with our observation of a strong myeloid infiltrate in Amela tumors [11] and a gene expression signature for Amela-infiltrating myeloid cells producing additional inflammatory cytokines (Table S2).

Evidence for the role of local inflammation in the promotion of melanoma progression has recently accumulated although the initiating events and the nature of the infiltrating cells may differ. For instance, ultraviolet B radiation (UVB), a major contributor to skin carcinogenesis, was shown to promote melanomagenesis in neonatal mice transgenic for hepatocyte growth factor (HGF) via recruitment of IFNγ-producing macrophages to neonatal skin by UVB-induced ligands to the chemokine receptor Ccr2 [40], [41], a process which does not occur in adult mice. Interestingly, HGF has been shown to induce epithelial scattering through MAPK-mediated upregulation of Snail1 [42].

In addition to this proinflammatory circuit in Amela tumors, we observed a gene expression signature characterizing an EMT-like process, which was not expressed by Mela tumors. Therefore a signaling link may exist between inflammatory cytokine gene expression and an EMT-like gene expression profile.

Amela melanomas showed a decrease in E-cadherin*/Cdh1* transcripts compared to Mela tumors. E-cadherin expression was decreased in melanoma cell lines compared to normal human epidermal melanocytes [43]. In contrast, transcripts for N-cadherin/*Cdh2*, another member of the cadherin family, were up-regulated in Amela cells. Loss of E-cadherin and gain of N-cadherin expression is known as “cadherin switching” [44]. It can promote motility and invasion of cancer cells and is usually observed in EMT, especially in melanoma [45].

Amela cells also expressed zinc-finger transcription factor Snail1, a transcriptional repressor of E-cadherin expression [46]. Expressed in many cancers, Snail1 was reported to regulate genes involved in EMT-like processes during malignant melanoma development [47]. TGFβ is a major inducer of Snail1 expression [48], associated with EMT [25], [49]. Our microarray results showed a higher expression of genes associated with TGFβ signaling in Amela compared to Mela tumors. Although not secreting active TGFβ, Amela cells appear to secrete the inactive form of TGFβ, which can be activated by different factors. We observed a constitutive activity of the TGFβ signaling pathway in Amela lines in the absence of exogenous TGFβ, which did not result from autocrine stimulation through TβRI (Fig. 3, Fig. S4A). Rather, it may depend on JNK activity which probably resulted from the activation of the Ras pathway in the Amela tumor lines, in agreement with studies on H-Ras^G12V^ transformed epithelial lines [7], [50] and patient colorectal tumors [50], [51]. The higher expression of H-Ras^G12V^ in the Amela as compared to the Mela tumors may thus contribute to activation of the Smad pathway leading to an EMT-like gene expression signature. Accordingly, expression of hallmark EMT genes such as *Snai1/Snail1* and *Fn1* was downregulated in the presence of a JNK inhibitor in conditions that increased expression of the *Cdh1* gene in Amela tumor lines (Fig. 4).

In melanoma, both the Ras-Raf-MEK-ERK (MAPK) and the PI3K-AKT (AKT) signaling pathways are constitutively activated through multiple mechanisms, and thus exert several key functions in melanoma development and progression [52]. In human melanomas expressing the BRAF mutation V600E, up-regulation of Snail1 was found to be inversely correlated with progression-free and overall survival of patients [53]. Its up-regulation appeared to depend mostly on ERK, but not on JNK activation in those melanomas. However, evidence for constitutive phosphor-JNK expression was also found in human melanoma lines expressing BRAF or NRAS activating mutations [54]. In the Amela tumors, JNK appeared to induce smad-3 activity and to contribute, together with activated ERK to expression of Snail1 and repression of Cdh1 gene expression. Constitutive production of the myeloid cell recruiting chemokine Ccl2 by the Amela cells also appeared dependent on both the JNK and the ERK activation pathways.

Of note, expression of another transcriptional regulator of the Snail-family, Slug a master regulator of neural crest cell specification and migration in many species [55], [56] encoded by the snai2 gene, was found to be down-modulated in the more aggressive Amela as compared to the Mela tumors (Fig. 1A – Tables S1). This observation is in line with data reporting higher expression of Slug in human benign melanocytic lesions prior to neoplastic transformation [55], [57] than in primary or metastatic melanomas in a manner that correlated with the level of expression of transcription factor Mitf [57] . Indeed, down-regulation of the pigmentation program in Amela tumor cells was associated with down- and up-regulation of Mitf and Brn2 transcription factors, respectively. In a recent study visualizing simultaneously increase of Brn2 promoter activity and decrease in pigmentation of melanoma cells, Brn2 expression was associated with a de-differentiated, invasive phenotype [58]. In a Braf mutant melanoma model in mice, increased Brn2 expression also led to increased invasiveness [59]. Among Brn2 target genes previously described in a melanoma line [60], some were up-regulated in Amela tumors and are known to play a role in invasiveness (*Hmga2*, *Twist1*, *Fn1)*. Interestingly many potential Brn2 target genes significantly up-regulated in Amela tumors are involved in the development of the nervous system linking the melanomas to their neural crest cell origin (data not shown).

For the Amela tumors the observation that more rapidly growing amelanotic tumors develop in some cases at the site where pigmented melanomas initially developed [10], [11] is in favor of a process of de-differentiation. *In vivo* transfer of primary melanotic tumors in immunodeficient mice also led to “outgrowth” of amelanotic tumors. Analysis at the clonal level should be performed to establish whether de-differentiation in pigmented cells gives rise to amelanotic cells or whether pre-existing amelanotic cells outgrew the pigmented cells. Since we generally observed only one tumor developing per mouse, the probability that two independent transformation events would be at the origin of the pigmented and amelanotic tumors at the same site is small. Therefore, a unique transformation event may have taken place in a precursor common to pigmented and non-pigmented cells of neuroectodermal origin, possibly at the level of a Schwann cell precursor [61]. Whether a non-pigmented cell constitutes the melanoma-initiating cell, as recently suggested [62], will require further studies. It is worth mentioning the potential importance of the notion that non-pigmented as well as pigmented cells may need to be targeted by therapeutic treatments. Indeed, immunotherapy protocols have often been directed at melanocyte differentiation antigens, which may not be expressed in non-pigmented tumor-precursor/-initiating/de-differentiated cells. In this respect the TiRP mouse melanoma model presents the advantage of being designed to express a known antigen concomitantly with the transforming oncogene. Ongoing pre-clinical immunotherapy protocols are testing the efficiency of treatments directed at such a model antigen [63].

## Materials and Methods

### Mice

#### Ethics statement

All procedures were approved by the Regional “Provence-Alpes-Cote d’Azur” Committee on Ethics for Animal Experimentation (authorization: #13.21, date: 11/02/2000) and were in accordance with French and European directives. All efforts were made to minimize animal suffering.

TiRP (*Tyr-iRas-P1A-transgenic Ink4a/Arf^flox/flox^*) mice [10], kept on the B10.D2 background [11] were treated as described [10], [11] for melanoma induction (Supplemental Information -SI- for details). Non-transgenic *Ink4a/Arf^flox/flox^* mice, used as controls, never develop melanomas.

### Mouse Melanoma Cell Lines

B16 mouse melanoma line B16F10 (ATCC number CRL-6475), originally derived by I. Fidler (M.D. Anderson Cancer Center, Houston, Tx), was received from Dr. AF Tilkin-Mariamé (INSERM-U563, Toulouse, France). Mela and Amela mouse melanoma cell lines were established in culture from, respectively, homogeneously melanotic and amelanotic induced melanomas in TiRP mice, as described [10] and were further cultured in DMEM (GibcoRL) with 10% FCS (see Text S1).

### Microarrays Analysis

Gene expression profiles were analyzed by two-color micro arrays on: (i) - induced Amela and Mela tumors (4 independent tumors of each type, being either homogeneously amelanotic (Amela) or homogeneously melanotic (Mela)) compared to each other or to control mice skin (pool from two control mice); (ii) - induced Amela tumors versus cultured lines established from induced Amela tumors (4 independent tumors). For each experimental sample, two technical replicates (dye-swaps) were examined.

### Isolation of RNA from Melanoma Tumors and Tumor Cell Lines

Total RNA was isolated from melanoma frozen tissues or skin (conserved at -80°C in RNA later) using RNeasy Mini Kit column purification and digestion with RNase free DNaseI according to the manufacturer’s protocols (Qiagen). Total RNA from cultured melanoma lines was extracted using Trizol reagent (Invitrogen life technologies Inc.) and resuspended in RNase-free water. RNA quality and quantity were determined using a bioanalyzer (Agilent technologies) and a Nanodrop spectrophotometer ND-1000 (Nanodrop technology), respectively.

### RNA Labeling and Hybridization

These steps were performed by the “Plate-Forme Transcriptome, Nice-Sophia Antipolis” as described [64] (see Text S1). Probe sequences are available on the MEDIANTE web site (http://www.microarray.fr:8080/merge/index).

### Data Analysis

Fluorescence intensity measurements and data analyses were as described (see Text S1). Microarray data have been submitted to NCBI GEO database: http://www.ncbi.nlm.nih.gov/geo/query/acc.cgi?acc=GSE29304.

### Quantitative Reverse Transcriptase Chain-reaction

cDNA was generated using the superscript first-strand synthesis system for RT-PCR according to the manufacturer’s instruction (Invitrogen). Quantitative real time PCR was performed with a Prism 7500 fast real time PCR system using Sybr green PCR Master Mix (Applied Biosystem). Thermal cycle conditions and primers are detailed (see Text S1).

### Active TGFβ Measurement

The MFBF11 TGF-reporter cells expressing a plasmid containing SMAD-binding elements driving the expression of secreted alkaline phosphatase (SEAP), kindly provided by Ina Tesseur (Stanford University School of Medicine, Stanford, CA), were treated as described [27]. Amela cells and reporter cells (MFBF11) were incubated overnight in 1 ml serum-free DMEM. 500 µl of the Amela cell supernatants were either acid-activated followed by neutralization to pH 7.4 or non-activated by addition of 100 µl NaCl 0.5 M (see Text S1). Treated supernatants were added to reporter cells. SEAP activity was measured using Great EscAPe SEAP Reporter system 3 (BD Biosciences, San Jose, CA) with a Lmax plate photometer (Molecular Devices, Sunnyvale, CA). The same assay was performed using reporter cell lines expressing the green fluorescent protein (SBE-GFP) [28]. Expression of GFP was measured by flow cytometry (CANTO II-BD Biosciences) and data were analyzed using FlowJo^TM^ software (Tree Star).

### Cell Transduction with Lentiviral Reporter Constructs for Fluorescence Tracking of Smad3 Signaling

Amela and B16F10 cell lines were transduced with Lentiviral vectors encoding the reporter SBE-GFP [28] (see Text S1). Infected cells were selected on the basis of GFP expression in the presence of TGFβ using a FACS.

### Flow Cytometry

SBE-GFP-transduced melanoma cells, incubated overnight at 1×10^5^ cells/well in 24-well flat-bottom tissue culture plates (BD Falcon, San Jose,CA), were washed twice with PBS and incubated in 1 ml serum-free DMEM/P/S for 24 h. After a further overnight incubation in the same medium or with TGFβ 10 ng/ml (recombinant human TGFβ; R&D), GFP expression was analyzed by flow cytometry (CANTO II-BD Biosciences) and data were analyzed using FlowJo^TM^ software (Tree Star).

### Kinase Inhibitors

SBE-GFP-transduced melanoma cells were treated as above except for the addition of inhibitors during the overnight incubation in the presence or absence of TGFβ 5 ng/ml. Inhibitors were Smad3 inhibitor SIS3 10 µM (Calbiochem), TGFβRI kinase inhibitor SB431542 10 µM, JNK inhibitor SP600125 10 µM (Calbiochem) and ERK inhibitor PD 98059 (10 µM). When testing the effect of kinase inhibitors on EMT or Ccl2 transcripts, Amela cells were plated at 10^5^ cells in 1 ml FCS containing medium for 24 h, followed by 24 h in medium without FCS and 24 h in the same medium in the presence of control DMSO or inhibitors as above. For each tumor line, cells from 4 wells were pooled and processed for QRT-PCR analysis.

### Immunofluorescence

Snap-frozen tumors in tissue-Tek (Sakura Finetek) were fixed in 4% paraformaldehyde, permeabilized with methanol and stained with a polyclonal rabbit anti-pSmad3L(Ser208/213) antibody (28029, IBL) or rabbit anti-pJNK antibody (ab4821, Abcam) and with anti-CD45 monoclonal antibody. Confocal microscopy was performed with a Zeiss LSM510 microscope. Image processing was performed with Zeiss LSM software and Adobe Photoshop.

### Detection of Ccl2 Transcripts by QRT-PCR and Secreted Protein by ELISA

Amela cell lines were cultured as indicated above in the absence or presence of kinase inhibitors. Culture supernatants were collected and kept frozen for detection of secreted Ccl2 by ELISA. The cell pellets were treated for detection of Ccl2 transcripts by QRT-PCR, as described above. Mouse Ccl2 (MCP-1) ELISA ready-SET-Go! (eBioscience) was used for quantification of Ccl2 production by Amela tumor lines.

### Statistical Analyses

Statistical analyses were performed with the Student’s unpaired *t* test using GraphPad and two-tailed *P* values are given as follows: (*) *P* < 0.05; (**) *P* < 0.01; and (***) *P* < 0.001.

## Supporting Information

Figure S1 **Unsupervised Hierarchical Clustering of melanoma tumors and healthy skin samples.** Each row represents a gene, and each column represents a sample. Each experimental sample is represented by 2 or 3 values associated to 2 or 3 different hybridizations. The expression level of each gene in a single tumor is relative to its median abundance across all tumors and is depicted according to a color scale in which red and green expression levels are, respectively, above and below the median. The magnitude of deviation from the median is represented by the color saturation. The dendrogram of samples (matrix on top) represents overall similarities in gene expression profiles. Four clusters are shown: - clusters of genes highly expressed selectively in Amela (A) or in Mela (B) tumors; - clusters of genes which expression is up-regulated (C) or down-regulated (D) in both tumors as compared to healthy skin.
(PDF)

Figure S2 **Heatmap output for the 80 most differentially expressed transcripts between Amela and Mela tumors.** Each row represents a gene and each column represents a sample. Each experimental sample is represented by 2 or 3 values associated to 2 or 3 different hybridizations. Expression values are represented as colors, where the range of colors (red, pink, light blue, dark blue) shows the range of expression values (high, moderate, low, lowest). These genes were provided in the GSEA plots shown in Fig. 2.
(PDF)

Figure S3 **EMT signature gene expression in Amela tumors.** Snail expression in Amela (A–C) and Mela (D–F) tumors was analyzed by immunohistology on tumor sections. It shows Dapi staining for nuclei (white), anti-CD45 antibody staining for leukocytes (green) and anti-Snail antibody staining (red). Scale bars: 50-µm (A, C, D, F) and 20-µm (B, E). In C and F, the anti-Snail antibody was pre-incubated with the immunizing peptide (see Supplemental Methods). Data are representative of 3 tumors of each type.
(PDF)

Figure S4 **Analysis of the TGFβ3 pathway in melanoma lines and tumors.** A. Supernatants from Amela^C^ and Amela^I^ cell lines incubated in serum-free DMEM were either acid treated (acid) or not (no treatment) and were tested for TGFβ using a reporter line expressing SBE-GFP (see Methods). The mean of GFP fluorescence intensity is represented. Bars represent means ± s.e.m. of triplicate wells for 4 samples in one representative experiment. Serum-free DMEM (no TGFβ) was used for baseline measurement. B. Control staining of Amela and Mela tumors analyzed by immunohistology in Fig. 4A in the presence of secondary goat anti-Rat fluorescent (Alexa546) antibody, but in the absence of Rat anti-Phospho-Smad3L antibody. Anti-CD45 mAb and Sytox blue staining are as in Fig. 4A.
(PDF)

Table S1 **Genes involved in pigmentation, differentiation and development of melanocytes down-regulated or up-regulated in Amela versus Mela tumors as shown in** **Figure 1A** **.**
(PDF)

Table S2 **Genes characterizing immune response components or chemotaxis that show higher expression in Amela vs Mela tumors but are expressed at lower level in Amela lines in culture than in Amela tumors.**
(PDF)

Table S3 **Table representing the genes highly expressed in Amela tumors having one or multiple conserved Smad binding sites in their promoter (in silico analysis).** For each gene, the conserved Smad binding sites (CAGA), their number and their p values are represented.
(PDF)
