# Supplementary figures and images for: Epithelial-Mesenchymal-Transition-Like and TGFβ Pathways Associated with Autochthonous Inflammatory Melanoma Development in Mice
Source: PLoS One. 2012 Nov 16;7(11):e49419. doi: 10.1371/journal.pone.0049419 (PMC3500287; doi:10.1371/journal.pone.0049419)

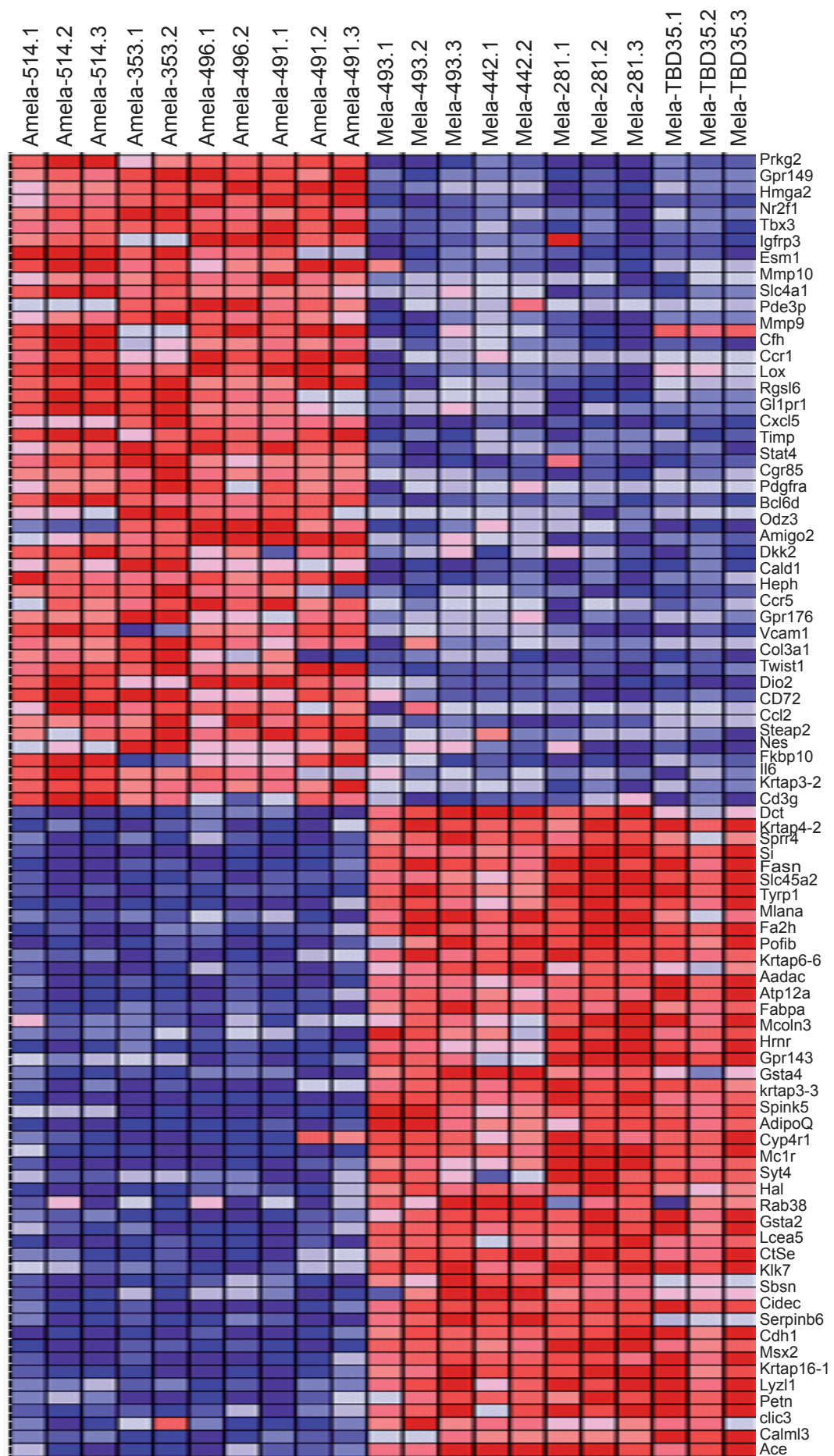

Figure S2

Supplement: Figure S2 — Heatmap output for the 80 most differentially expressed transcripts between Amela and Mela tumors. Each row represents a gene and each column represents a sample. Each experimental sample is represented by 2 or 3 values associated to 2 or 3 different hybridizations. Expression values are represented as colors, where the range of colors (red, pink, light blue, dark blue) shows the range of expression values (high, moderate, low, lowest). These genes were provided in the GSEA plots shown in Fig. 2. (PDF) [file pone.0049419.s002.pdf]

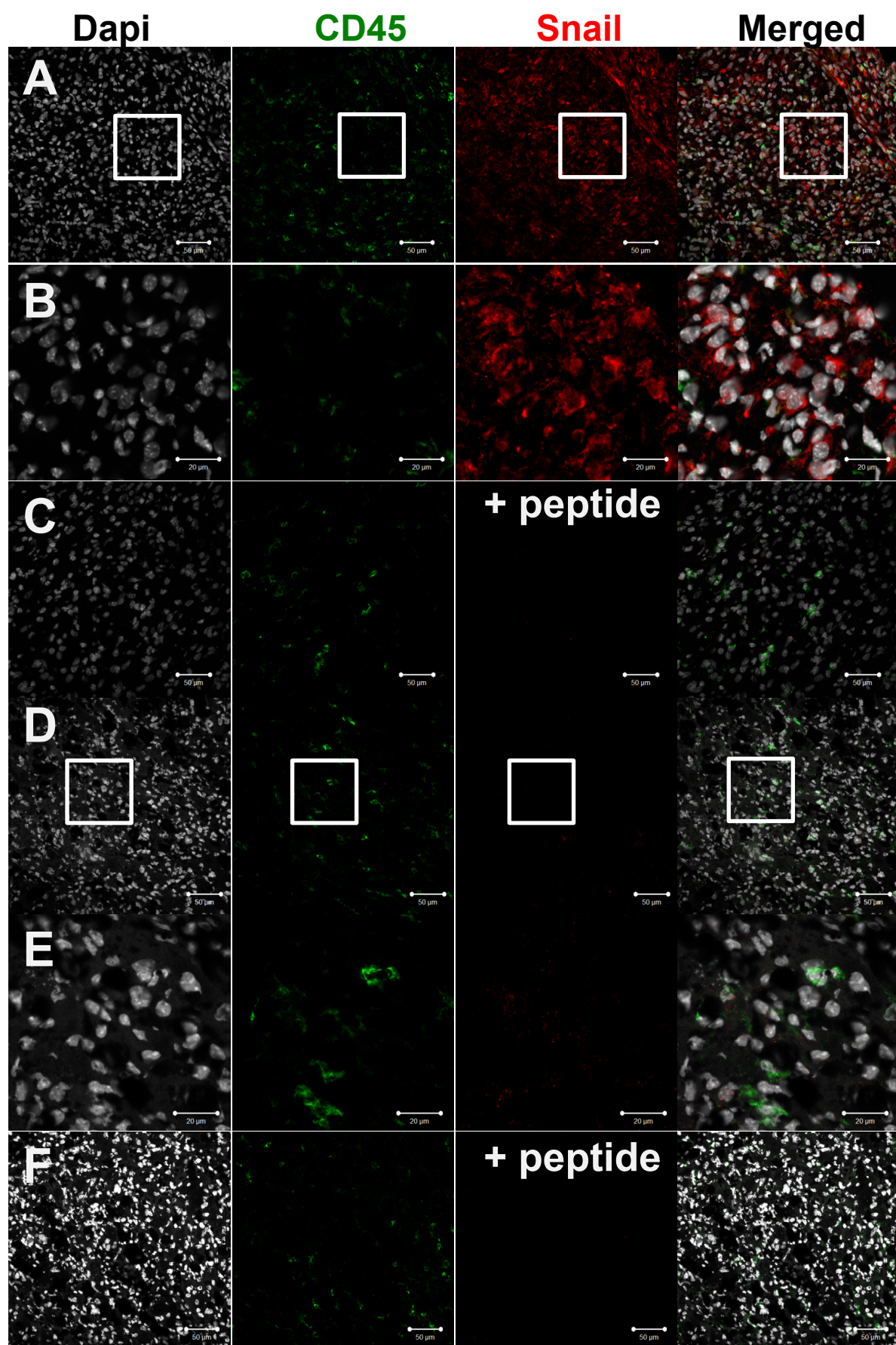

Amela

Mela

**Figure S3**

Supplement: Figure S3 — EMT signature gene expression in Amela tumors. Snail expression in Amela (A–C) and Mela (D–F) tumors was analyzed by immunohistology on tumor sections. It shows Dapi staining for nuclei (white), anti-CD45 antibody staining for leukocytes (green) and anti-Snail antibody staining (red). Scale bars: 50-µm (A, C, D, F) and 20-µm (B, E). In C and F, the anti-Snail antibody was pre-incubated with the immunizing peptide (see Supplemental Methods). Data are representative of 3 tumors of each type. (PDF) [file pone.0049419.s003.pdf]

**A**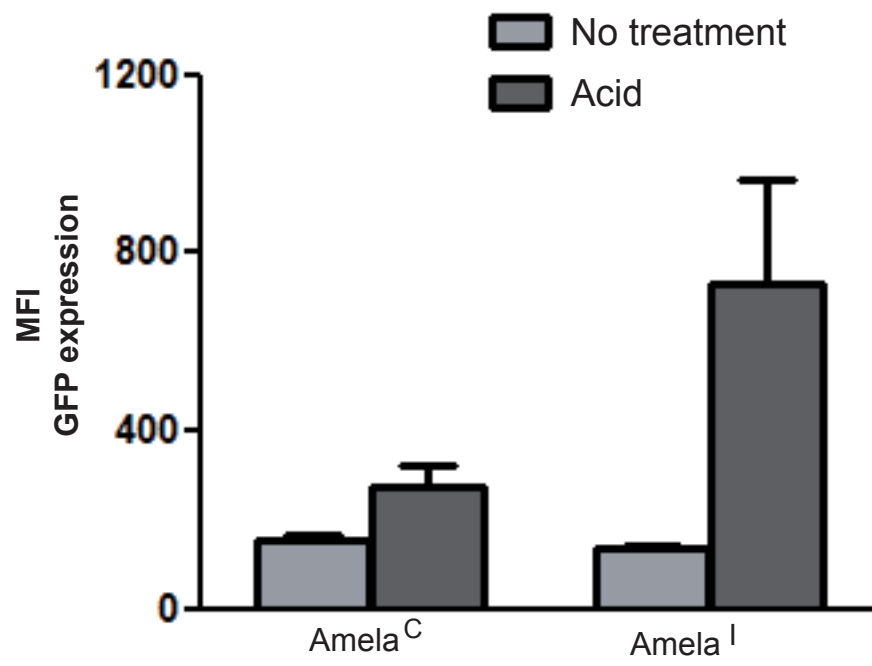**B**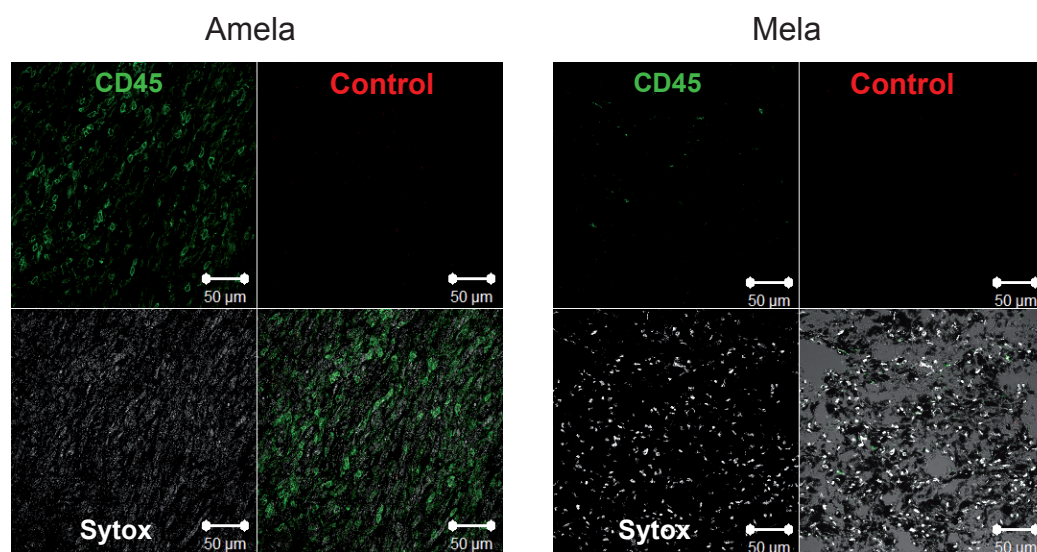

Figure S4

Supplement: Figure S4 — Analysis of the TGFβ3 pathway in melanoma lines and tumors. A. Supernatants from AmelaC and AmelaI cell lines incubated in serum-free DMEM were either acid treated (acid) or not (no treatment) and were tested for TGFβ using a reporter line expressing SBE-GFP (see Methods). The mean of GFP fluorescence intensity is represented. Bars represent means ± s.e.m. of triplicate wells for 4 samples in one representative experiment. Serum-free DMEM (no TGFβ) was used for baseline measurement. B. Control staining of Amela and Mela tumors analyzed by immunohistology in Fig. 4A in the presence of secondary goat anti-Rat fluorescent (Alexa546) antibody, but in the absence of Rat anti-Phospho-Smad3L antibody. Anti-CD45 mAb and Sytox blue staining are as in Fig. 4A. (PDF) [file pone.0049419.s004.pdf]
