# Supplementary material for: Epithelial-Mesenchymal-Transition-Like and TGFβ Pathways Associated with Autochthonous Inflammatory Melanoma Development in Mice
Source: PLoS One. 2012 Nov 16;7(11):e49419. doi: 10.1371/journal.pone.0049419 (PMC3500287; doi:10.1371/journal.pone.0049419)
Supplement: Table S1 — Genes involved in pigmentation, differentiation and development of melanocytes down-regulated or up-regulated in Amela versus Mela tumors as shown in Figure 1A . (PDF) [file pone.0049419.s005.pdf]

Supplemental information

Supplemental methods

Mice

TiRP-10B;Ink4a/Arf flox/flox mice (Huijbers et al 2006) backcrossed to the B10.D2 strain (B10.D2/nOlaHsd, H-2d) Harlan (Gannat, France)) for more than 10 generations and hereafter called TiRP mice were previously described (Soudja et al 2010). TiRP mice devoid of adaptive immune components were established after crossing with Rag-1 KO B10.D2 (RagKO) mice (Soudja et al 2010), and are hereafter called TiRP RagKO mice. Treatment of TiRP mice with 4OH-tamoxifen (Sigma) was as previously described (Huijbers et al 2006). Mice were housed under specific pathogen-free conditions.

Melanoma cell lines

Established in culture from Amela melanomas developing in TiRP and TiRP RagKO mice as described (Huijbers et al 2006), melanoma cell lines were further cultured in DMEM complete medium (GibcoRL), supplemented with 10% FCS, enriched with glutamine, penicillin-streptomycin, HEPES and Sodium pyruvate.

RNA labeling and hybridization

These steps were performed by the "Plate-Forme Transcriptome, Nice-Sophia Antipolis" under direction of Dr. Pascal Barbry (CNRS-UMR6097, Nice-Sophia-Antipolis, France) as described (Le Brigand et al 2006). Briefly, 1μg total RNA was amplified and labeled with Cy3 and Cy5 fluorochromes using the Amino Allyl MessageAmp aRNA kit according to the manufacturer's (Ambion) protocol. Cy3-and Cy5-labeled cRNAs were fragmented using Fragmentation Buffer (Agilent), dissolved in Hybridization Buffer (Agilent) and hybridized to pan-genomic mouse micro arrays of the RNG/MRC resource (http://www.microarray.fr). These micro arrays harbor 24109 50mer oligonucleotides (Le Brigand et al 2006).

Data analysis

Fluorescence intensity measurements were subjected to automatic background subtraction. The Cy3/Cy5 ratios were normalized to the value of the median ratio of all spots in the array and bad spots were excluded from the analysis, as described (Le Brigand et al 2006). All ratio values were log-transformed (base 2), and duplicated spots in the array were averaged. A single value for each gene was obtained. For our purposes, expression of a gene was deemed to be up regulated or down regulated if there was at least a 2-fold difference in the expression and if the P value was <0.001. The regulated genes were functionally classified manually on the basis of exhaustive searches in Pubmed and gene ontology. The open source R software

package (http://www.r-project.org) and tools from the Bioconductor project (http://www.bioconductor.org) Gene Cluster, TreeView, and Excel were used for processing and analysis of the micro array data. Data from each hybridization were maintained in a database for analysis.

Quantitative reverse transcriptase chain-reaction

cDNA was generated using the superscript first-strand synthesis system for RT-PCR according to the manufacturer's instruction (Invitrogen). Quantitative real time PCR was performed with a Prism 7500 fast real time PCR system using Sybr green PCR Master Mix (Applied Biosystem). The thermal cycle conditions were as follows: hold for 10 min at 95° C followed by two steps PCR for 40 cycles at 95°C for 15 s and at 60°C for 1 min. Optimal annealing temperature was predetermined to ensure single amplified product. All samples were performed in duplicate. The β-actin gene was used as control. Threshold cycle (Ct) was assigned to various products according to the cycle number at which fixed, low fluorescence intensity was achieved. ΔCt = Ct (target) - Ct (actin), the difference between the Ct value of the target gene and the Ct value of the reference gene (actin). The fold increase of the target gene mRNA was calculated according to the expression 2-ΔΔCt, where 2-ΔΔCt is the difference between ΔC (experimental sample) and ΔC (control sample).

Primers:

Gene		Primer sequence 5'---3'	Product size (bp)
β-Actin	F[a]:	GACGGCCAGGTCATCACTATTG	72
	B[b]:	CAAGAAGGAAGGCTGGAAAAGA	
E-Cadherin	F:	GTCAGATCTCCCTGAGTTCG	392
	B:	GCACCCACACACATACACTC	
Brn2	F:	CGGCGGTTTGCTCTATTC	118
	B:	ATGGTGTGGCTCATCGTG	
Hmga2	F:	AGCAAAAACAAGAGCCCCTCTA	94
	B:	ACGACTTGTTGTGGCCATTTC	
Id2	F:	AAAACAGCCTGTCGGACCAC	121
	B:	CTGGGCACCAGTTCCTTGAG	
Snail	F:	ACCCCCGCCGGAAGCCCAACT	128
	B:	AGCGGCGGGGTTGAGGACCTC	
Twist	F:	CGGGTCATGGCTAACGTG	243
	B:	CAGCTTGCCATCTTGGAGTC	

Mitf	F:	GCCTTGTTTATGGTGCCTTC	73
	B:	GTCCTCCTCCCTCTACTTTCTGT	

a: F:forward b: B:Backward

Active TGFβ measurement

The MFBF11 TGF-reporter cells expressing a plasmid containing SMAD-binding elements driving the expression of secreted alkaline phosphatase (SEAP), kindly provided by Ina Tesseur (Stanford University School of Medicine, Stanford, CA), were treated as described (Tesseur et al. 2006). Amela cells and reporter cells (MFBF11) were seeded at $1x10^5$ cells/well in 24-well flat-bottom tissue culture plates (BD Falcon, San Jose, CA). After overnight incubation, cells were washed twice with PBS and incubated in 1 ml serum-free DMEM supplemented with Penicillin/streptomycin (DMEM/P/S). 500µl of these Amela cells supernatant were either acid-activated by adding 50µl 1M HCl at room temperature for 10min followed by neutralization to pH 7.4 with 1M NaOH or non-activated by addition of 100µl NaCl 0.5M. Treated supernatants were added to reporter cells. SEAP activity was measured using Great EscAPe SEAP Reporter system 3 (BD Biosciences, San Jose, CA) with a Lmax plate photometer (Molecular Devices, Sunnyvale, CA). The same assay was performed using reporter cell lines expressing the green fluorescent protein (SBE-GFP) (Stuelten et al. 2007). Expression of GFP was measured by flow cytometry (CANTO II-BD Biosciences) and data were analyzed using FlowJo™ software (Tree Star).

Cell transduction with lentiviral reporter constructs for fluorescence tracking of Smad3 signaling

Amela and B16 cell lines were transduced with Lentiviral vectors encoding the reporter SBE-GFP (Stuelten et al 2007), kindly provided by Dr. Christina Stuelten (NCI, NIH, Bethesda, MD). Cells were seeded at $1x10^4$ cells/well in 96-well flat-bottom tissue culture plates, lentiviral particles were added to the cells in the presence of polybrene (o.8mg/ml). Infected cells were selected on the basis of GFP expression in the presence of TGFβ using a FACS.

Supplemental legend to Table 3.

References corresponding to Table 3:

1. Untergasser, G., et al., *Profiling molecular targets of TGF-beta1 in prostate fibroblast-to-myofibroblast transdifferentiation.* Mech Ageing Dev, 2005. **126**(1): p. 59-69.
2. Kapoun, A.M., et al., *Transforming growth factor-beta receptor type 1 (TGFbetaRI) kinase activity but not p38 activation is required for TGFbetaRI-induced myofibroblast differentiation and profibrotic gene expression.* Mol Pharmacol, 2006. **70**(2): p. 518-31.
3. Venkatesan, N., P.J. Roughley, and M.S. Ludwig, *Proteoglycan expression in bleomycin lung fibroblasts: role of transforming growth factor-beta(1) and interferon-gamma.* Am J Physiol Lung Cell Mol Physiol, 2002. **283**(4): p. L806-14.
4. Verrecchia, F., M.L. Chu, and A. Mauviel, *Identification of novel TGF-beta /Smad gene targets in dermal fibroblasts using a combined cDNA microarray/promoter transactivation approach.* J Biol Chem, 2001. **276**(20): p. 17058-62.
5. Gaspar, N.J., et al., *Inhibition of transforming growth factor beta signaling reduces pancreatic adenocarcinoma growth and invasiveness.* Mol Pharmacol, 2007. **72**(1): p. 152-61.
6. Valcourt, U., et al., *TGF-beta and the Smad signaling pathway support transcriptomic reprogramming during epithelial-mesenchymal cell transition.* Mol Biol Cell, 2005. **16**(4): p. 1987-2002.
7. Xie, L., et al., *Transforming growth factor beta-regulated gene expression in a mouse mammary gland epithelial cell line.* Breast Cancer Res, 2003. **5**(6): p. R187-98.
8. Saharinen, J., et al., *Latent transforming growth factor-beta binding proteins (LTBPs)--structural extracellular matrix proteins for targeting TGF-beta action.* Cytokine Growth Factor Rev, 1999. **10**(2): p. 99-117.
9. Dong, Y.W., X.P. Wang, and K. Wu, *Suppression of pancreatic carcinoma growth by activating peroxisome proliferator-activated receptor gamma involves angiogenesis inhibition.* World J Gastroenterol, 2009. **15**(4): p. 441-8.
10. Ortiz, C.O., et al., *Transforming growth factor-beta regulation of the insulin-like growth factor binding protein-4 protease system in cultured human osteoblasts.* J Bone Miner Res, 2003. **18**(6): p. 1066-72.
11. Douthwaite, J.A., et al., *Effects of transforming growth factor-beta1 on renal extracellular matrix components and their regulating proteins.* J Am Soc Nephrol, 1999. **10**(10): p. 2109-19.
12. Zavadil, J., et al., *Genetic programs of epithelial cell plasticity directed by transforming growth factor-beta.* Proc Natl Acad Sci U S A, 2001. **98**(12): p. 6686-91.
13. Levy, L. and C.S. Hill, *Smad4 dependency defines two classes of transforming growth factor {beta} (TGF-{beta}) target genes and distinguishes TGF-{beta}-induced epithelial-mesenchymal transition from its antiproliferative and migratory responses.* Mol Cell Biol, 2005. **25**(18): p. 8108-25.
14. Kowanetz, M., et al., *Id2 and Id3 define the potency of cell proliferation and differentiation responses to transforming growth factor beta and bone morphogenetic protein.* Mol Cell Biol, 2004. **24**(10): p. 4241-54.
15. Cabello-Verrugio, C. and E. Brandan, *A novel modulatory mechanism of transforming growth factor-beta signaling through decorin and LRP-1.* J Biol Chem, 2007. **282**(26): p. 18842-50.

16. Hu, K., et al., *Tissue-type plasminogen activator promotes murine myofibroblast activation through LDL receptor-related protein 1-mediated integrin signaling.* J Clin Invest, 2007. **117**(12): p. 3821-32.
17. Ge, G. and D.S. Greenspan, *BMP1 controls TGFbeta1 activation via cleavage of latent TGFbeta-binding protein.* J Cell Biol, 2006. **175**(1): p. 111-20.
18. Salazar, K.D., S.M. Lankford, and A.R. Brody, *Mesenchymal stem cells produce Wnt isoforms and TGF-beta1 that mediate proliferation and procollagen expression by lung fibroblasts.* Am J Physiol Lung Cell Mol Physiol, 2009. **297**(5): p. L1002-11.
19. Massague, J., *TGFbeta in Cancer.* Cell, 2008. **134**(2): p. 215-30.
20. Dzwonek, J., et al., *Smad3 is a key nonredundant mediator of transforming growth factor beta signaling in Nme mouse mammary epithelial cells.* Mol Cancer Res, 2009. **7**(8): p. 1342-53.
21. Abboud, S.L., *Regulation of platelet-derived growth factor A and B chain gene expression in bone marrow stromal cells.* J Cell Physiol, 1995. **164**(2): p. 434-40.
22. Berking, C., et al., *Transforming growth factor-beta1 increases survival of human melanoma through stroma remodeling.* Cancer Res, 2001. **61**(22): p. 8306-16.
23. Katabami, K., et al., *Transforming growth factor-beta1 upregulates transcription of alpha3 integrin gene in hepatocellular carcinoma cells via Ets-transcription factor-binding motif in the promoter region.* Clin Exp Metastasis, 2005. **22**(7): p. 539-48.
24. Kuo, Y.C., et al., *Transforming growth factor-beta induces CD44 cleavage that promotes migration of MDA-MB-435s cells through the up-regulation of membrane type 1-matrix metalloproteinase.* Int J Cancer, 2009. **124**(11): p. 2568-76.

Supplemental figure legends.

Figure S1. Unsupervised Hierarchical Clustering of melanoma tumors and healthy skin samples.

Each row represents a gene, and each column represents a sample. Each experimental sample is represented by 2 or 3 values associated to 2 or 3 different hybridizations. The expression level of each gene in a single tumor is relative to its median abundance across all tumors and is depicted according to a color scale in which red and green expression levels are, respectively, above and below the median. The magnitude of deviation from the median is represented by the color saturation. The dendrogram of samples (matrix on top) represents overall similarities in gene expression profiles. Four clusters are shown: - clusters of genes highly expressed selectively in Amela (A) or in Mela (B) tumors; - clusters of genes which expression is up-regulated (C) or down-regulated (D) in both tumors as compared to healthy skin.

Figure S2. Heatmap output for the 80 most differentially expressed transcripts between Amela and Mela tumors.

Each row represents a gene and each column represents a sample. Each experimental sample is represented by 2 or 3 values associated to 2 or 3 different hybridizations. Expression values are represented as colors, where the range of colors (red, pink, light blue, dark blue) shows the range of expression values (high, moderate, low, lowest). These genes were provided in the GSEA plots shown in Fig.2.

Figure S3. EMT signature gene expression in Amela tumors.

Snail expression in Amela and Mela tumors was analyzed by immunohistology on ~~ex-vivo~~ tumors sections. It shows Topro-3 blue staining for nuclei (Blue) and anti-Snail antibody staining (red) together with anti-CD45 antibody staining for leukocytes (green). Scale bars: 10-µm.

Figure S4. Analysis of the TGFb3 pathway in melanoma lines and tumors.

A. Supernatants from Amela^{C} and Amela^{I} cell lines incubated in serum-free DMEM were either acid treated (acid) or not (no treatment) and were tested for TGFb using a reporter line expressing SBE-GFP (see Methods). The mean of GFP fluorescence intensity is represented. Bars represent means ± s.e.m. of triplicate wells in one representative experiment. Serum-free DMEM (no TGF-β) was used for baseline measurement. B. Control staining of Amela and Mela tumors analyzed by immunohistology in Fig.4A in the presence of secondary goat anti-Rat fluorescent (Alexa546) antibody, but in the absence of Rat anti-Phospho-Smad3L antibody. Anti-CD45 mAb and Sytox blue staining are as in Fig.4A.

Table S1. Genes involved in pigmentation, differentiation and development of melanocytes down-regulated or up-regulated in Amela versus Mela tumors as shown in Figure 1A.

Table S2. Genes characterizing immune response components or chemotaxis that show higher expression in Amela vs Mela tumors but are expressed at lower level in Amela lines in culture than in Amela tumors.

Table S3. Table representing the genes highly expressed in Amela tumors having one or multiple conserved Smad binding sites in their promoter (in silico analysis). For each gene, the conserved Smad binding sites (CAGA), their number and their p values are represented.

Table S1. Genes involved in pigmentation, differentiation and development of melanocytes differentially expressed by Mela versus Amela tumors.

Gene Symbol	Gene name	[b]Log2 ratio Amela/Mela
[a] Up-regulated in Mela		
Dct	Dopachrome tautomerase	-5.985
Sox9	SRY-box containing gene 9	-5.646
Tyrp1	Tyrosinase related protein 1	-5.392
Si	Silver	-5.373
Mlana	Melan-A	-5.262
Slc45a2	Solute carrier family 45, member 2	-4.564
S100b	S100 calcium binding protein B	-3.506
Mc1r	Melanocortin 1 receptor	-3.027
Mcoln3	Mucolipin 3	-2.976
Mitf	Microphtalmia transcription factor	-2.310
Sox10	SRY-box containing gene 10	-2.095
Pax3	Paired box gene 3	-2.045
Kit	Kit oncogene	-1.808
Mlph	Melanophilin	-1.412
Psen2	Presenilin 2	-1.408
Snai2	Snail homolog 2 (drosophila)	-0.897*
Erbb2	V-erb-b2 erythroblastic leukemia viral oncogene homolog 2	-0.755*
[c]Up-regulated in Amela		
Pou3f2	POU domain, class 3, transcription factor 2	1.31

(a) Genes involved in pigmentation. differentiation and development of melanocytes that show higher expression in Mela versus Amela ex vivo tumors
(b) Ratio of gene expression as log2 Amela/Mela < -1 with p values < 0.05;
* Ratio of gene expression as log2 Amela/Mela between -1 and 0 with p values < 0.05
(c) Ratio of gene expression as log2 Amela/Mela > 1 with p value < 0.05.

Table S2. Amela tumor-expressed genes characterizing tumor-infiltrating leukocytes.

Gene symbol	Gene name	[b]Log2 ratio Amela/Line	[c]Log2 ratio Amela/Mela
[a]*Immune response and chemotaxis*			
Emr1	EGF-like module containing, mucin-like, hormone receptor-like sequence 1	5.94	0.13
Ly86	Lymphocyte antigen 86	5.72	1.09
Mrc1	Mannose receptor, c type 1	5.60	1.12
Fcgr3	Fc gamma receptor 3	4.86	0.84*
Fbn1	Fibrilin 1	4.77	1.16
Msr2	Fc receptor-like S, scavenger receptor	4.60	1.12
Ccl12	Chemokine (C-C motif) ligand 12	4.19	1.71
Mmp3	Matrix metallopeptidase 3	4.18	2.05
Cd72	CD72 antigen	4.10	1.85
C1qa	Complement component 1, q subcomponent, A chain	4.08	1.39
Cx3cr1	Chemokine (C-X3-C) receptor 1	3.90	1.51
Mmp23	Matrix metallopeptidase 23	3.62	1.27
C1qr1	CD93 antigen	3.35	1.81
Csf1r	Colony stimulating factor 1 receptor	2.93	1.10
Slamf9	SLAM family member 9	2.86	1.05
Ltbp2	Latent transforming growth factor beta binding protein 2	2.78	1.16
Ccr1	Chemokine (C-C motif) receptor 1	2.61	2.07
Tgfbr2	Transforming growth factor, beta receptor II	2.24	0.96*
Lsp1	Lymphocyte specific 1	2.19	1.28
Tlr1	Toll like receptor 1	2.18	1.18
Cfh	Complement component factor h	2.01	2.15
Ltbp3	Latent transforming growth factor beta binding protein 3	1.57	0.96*
Mmp2	Matrix metallopeptidase 2	1.34	0.91*

(a) Genes characterizing immune response components or chemotaxis that show higher expression in Amela versus Mela ex vivo tumors but are expressed at lower level in Amela lines in culture than in Amela ex vivo tumors.
(b) Ratio of gene expression as Log2 Amela/Cultured Amela line > 1 with p value < 0.001;
(c) Ratio of gene expression as Log2 Amela/Mela > 1 with p value < 0.001;
* Ratio of gene expression as log2 Amela/Mela between 0 and 1 with p values < 0.05.

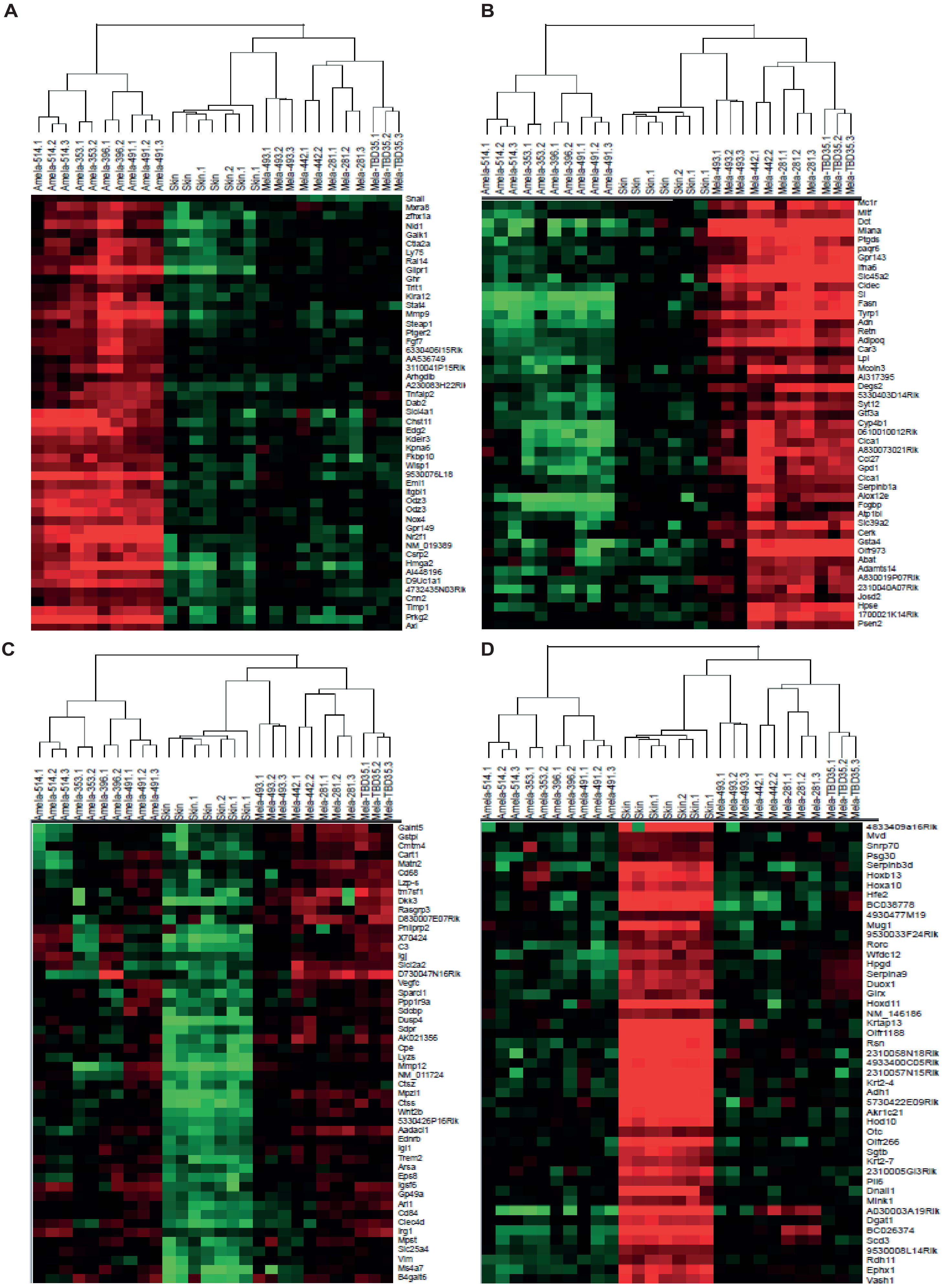
A
Amela-514.1
Amela-514.2
Amela-514.3
Amela-353.1
Amela-353.2
Amela-396.1
Amela-396.2
Amela-491.1
Amela-491.2
Amela-491.3
Skin
Skin
Skin.1
Skin
Skin.2
Skin.1
Skin.1
Mela-493.1
Mela-493.2
Mela-493.3
Mela-442.1
Mela-442.2
Mela-281.1
Mela-281.2
Mela-281.3
Mela-TBD35.1
Mela-TBD35.2
Mela-TBD35.3
Snail
Mxra8
zfhx1a
Nid1
Galk1
Ctla2a
Ly75
Ral14
Glipr1
Ghr
Tnt1
Klra12
Stat4
Mmp9
Steap1
Ptger2
Fgf7
6330406I15Rik
AA536749
3110041P15Rik
Arhgdib
A230083H22Rik
Tnfaip2
Dab2
Slc4a1
Chst11
Edg2
Kdelr3
Kpna6
Fkbp10
Wisp1
9530076L18
Emil1
Itgbl1
Odz3
Odz3
Nox4
Gpr149
Nr2f1
NM_019389
Csrp2
Hmga2
AI448196
D9Ucla1
4732435N03Rik
Cnn2
Timp1
Prkg2
Axl
B
Amela-514.1
Amela-514.2
Amela-514.3
Amela-353.1
Amela-353.2
Amela-396.1
Amela-396.2
Amela-491.1
Amela-491.2
Amela-491.3
Skin
Skin
Skin.1
Skin
Skin.2
Skin.1
Skin.1
Mela-493.1
Mela-493.2
Mela-493.3
Mela-442.1
Mela-442.2
Mela-281.1
Mela-281.2
Mela-281.3
Mela-TBD35.1
Mela-TBD35.2
Mela-TBD35.3
Mc1r
Mitf
Dct
Mlana
Ptgds
paqr6
Gpr143
Itna6
Slc45a2
Cidec
Sl
Fasn
Tyrp1
Adn
Retn
Adipoq
Car3
Lpl
Mcoln3
AI317395
Degs2
5330403D14Rik
Syt12
Gtf3a
Cyp4b1
0610010012Rik
Clca1
A830073021Rik
Ccl27
Gpd1
Clca1
Serpinb1a
Alox12e
Fcgbp
Atp1bl
Slc39a2
Cerk
Gsta4
Olfr973
Abat
Adamts14
A830019P07Rik
2310040A07Rik
Josd2
Hpse
1700021K14Rik
Psen2
C
Amela-514.1
Amela-514.2
Amela-514.3
Amela-353.1
Amela-353.2
Amela-396.1
Amela-396.2
Amela-491.1
Amela-491.2
Amela-491.3
Skin
Skin
Skin.1
Skin
Skin.2
Skin.1
Skin.1
Mela-493.1
Mela-493.2
Mela-493.3
Mela-442.1
Mela-442.2
Mela-281.1
Mela-281.2
Mela-281.3
Mela-TBD35.1
Mela-TBD35.2
Mela-TBD35.3
Galnt5
Gstpi
Cmtm4
Cart1
Matn2
Cd68
Lzp-s
Tm7sf1
Dkk3
Rasgrp3
D830007E07Rik
Pnliprp2
X70424
C3
Igj
Slc2a2
D730047N16Rik
Vegfc
Sparcl1
Ppp1r9a
Sdcbp
Dusp4
Sdpr
AK021356
Cpe
Lyzs
Mmp12
NM_011724
Ctsz
Mpzl1
Ctss
Wnt2b
5330426P16Rik
Aadacl1
Ednrb
Igf1
Trem2
Arsa
Eps8
Igsf6
Gp49a
Arl1
Cd84
Clec4d
Irg1
Mpst
Slc25a4
Vim
Ms4a7
B4galt6
D
Amela-514.1
Amela-514.2
Amela-514.3
Amela-353.1
Amela-353.2
Amela-396.1
Amela-396.2
Amela-491.1
Amela-491.2
Amela-491.3
Skin
Skin
Skin.1
Skin
Skin.2
Skin.1
Skin.1
Mela-493.1
Mela-493.2
Mela-493.3
Mela-442.1
Mela-442.2
Mela-281.1
Mela-281.2
Mela-281.3
Mela-TBD35.1
Mela-TBD35.2
Mela-TBD35.3
4833409a16Rik
Mvd
Snrp70
Psg30
Serpinb3d
Hoxb13
Hoxa10
Hfe2
BC036778
4930477M19
Mug1
9530033F24Rik
Rorc
Wfdc12
Hpgd
Serpina9
Duox1
Glrx
Hoxd11
NM_146186
Krtap13
Olfr1188
Rsn
2310058N18Rik
4933400C05Rik
2310057N15Rik
Krt2-4
Adh1
5730422E09Rik
Akr1c21
Hod10
Otc
Olfr266
Sgtb
Krt2-7
2310005G13Rik
Pli6
Dnall1
Mink1
A030003A19Rik
Dgat1
BC026374
Scd3
9530008L14Rik
Rdh11
Ephx1
Vash1

Figure S1

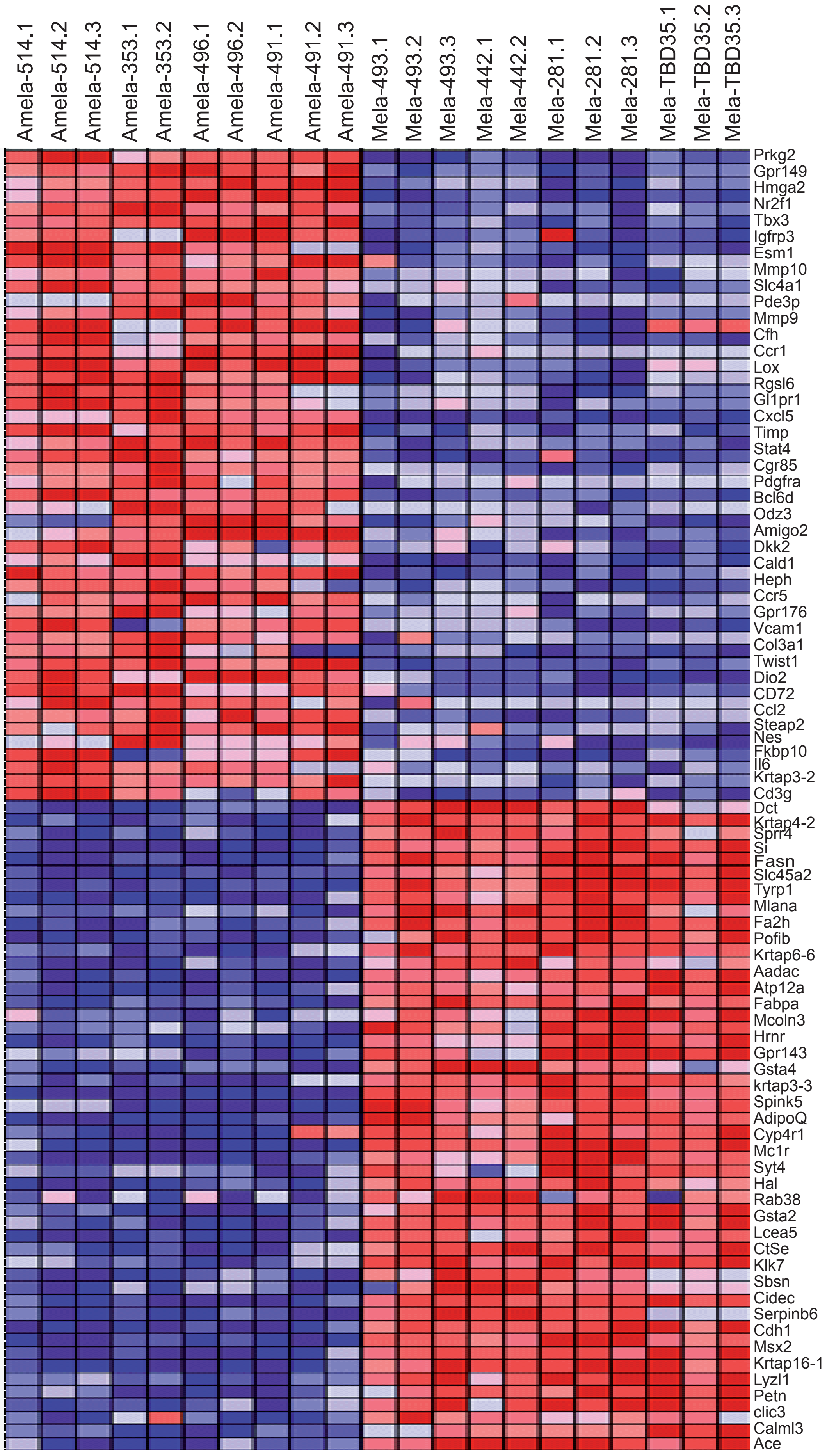
Amela-514.1
Amela-514.2
Amela-514.3
Amela-353.1
Amela-353.2
Amela-496.1
Amela-496.2
Amela-491.1
Amela-491.2
Amela-491.3
Mela-493.1
Mela-493.2
Mela-493.3
Mela-442.1
Mela-442.2
Mela-281.1
Mela-281.2
Mela-281.3
Mela-TBD35.1
Mela-TBD35.2
Mela-TBD35.3
Prkg2
Gpr149
Hmga2
Nr2f1
Tbx3
Igfrp3
Esm1
Mmp10
Slc4a1
Pde3p
Mmp9
Cfh
Ccr1
Lox
Rgsl6
Gl1pr1
Cxcl5
Timp
Stat4
Cgr85
Pdgfra
Bcl6d
Odz3
Amigo2
Dkk2
Cald1
Heph
Ccr5
Gpr176
Vcam1
Col3a1
Twist1
Dio2
CD72
Ccl2
Steap2
Nes
Fkbp10
Il6
Krtap3-2
Cd3g
Dct
Krtap4-2
Sprr4
Si
Fasn
Slc45a2
Tyrp1
Mlana
Fa2h
Pofib
Krtap6-6
Aadac
Atp12a
Fabpa
Mcoln3
Hrnr
Gpr143
Gsta4
krtap3-3
Spink5
AdipoQ
Cyp4r1
Mc1r
Syt4
Hal
Rab38
Gsta2
Lcea5
CtSe
Klk7
Sbsn
Cidec
Serpinb6
Cdh1
Msx2
Krtap16-1
Lyzl1
Petn
clic3
Calml3
Ace

Figure S2

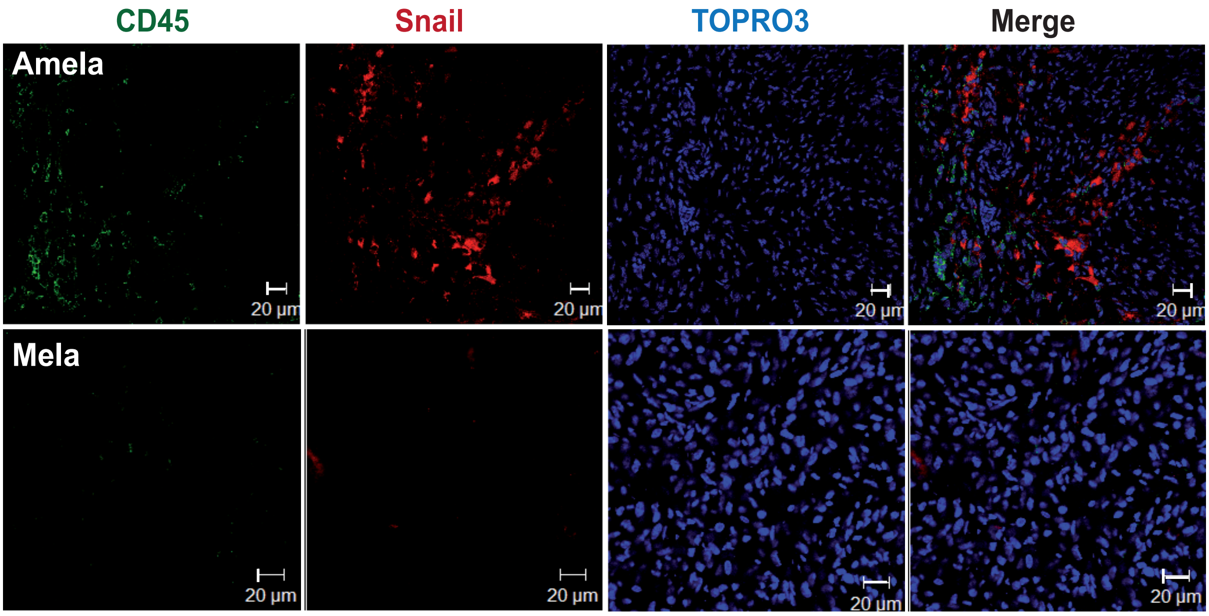

Figure S3

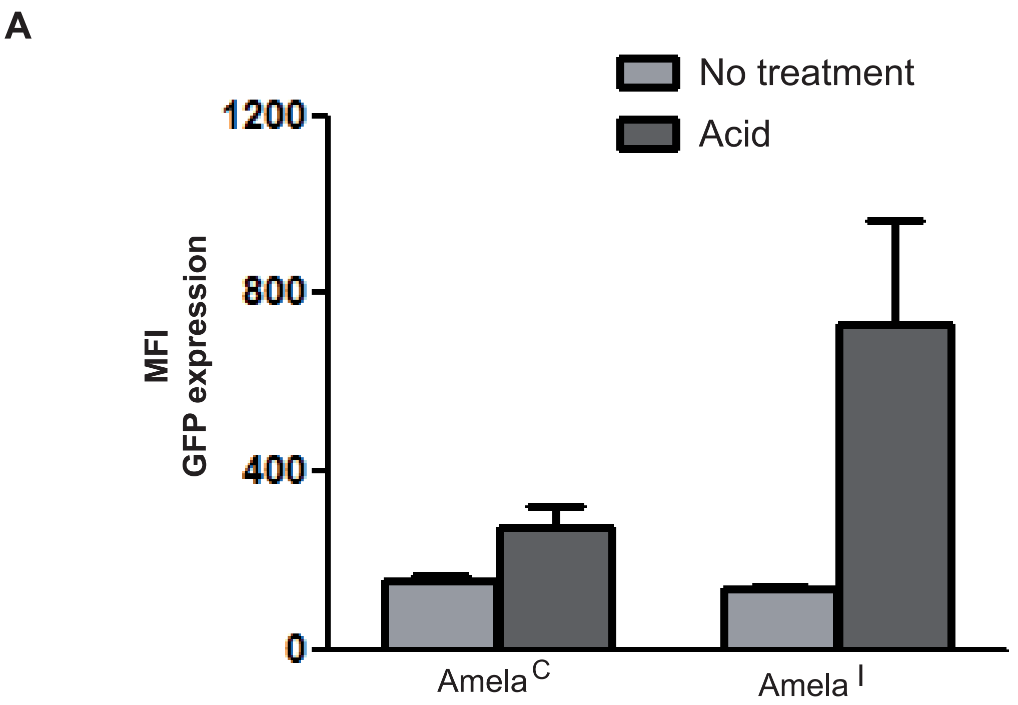
A
No treatment
Acid
MFI
GFP expression
1200
800
400
0
Amela C
Amela I

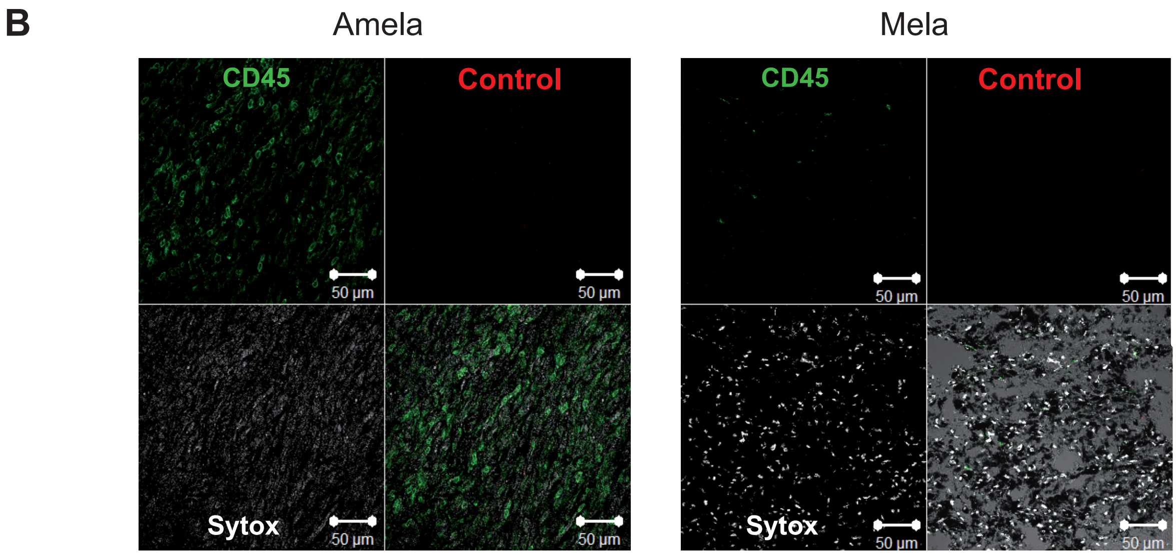
B
Amela
Mela
CD45
Control
Sytox
50 µm

Figure S4
