# Supplementary material for: Epithelial-Mesenchymal-Transition-Like and TGFβ Pathways Associated with Autochthonous Inflammatory Melanoma Development in Mice
Source: PLoS One. 2012 Nov 16;7(11):e49419. doi: 10.1371/journal.pone.0049419 (PMC3500287; doi:10.1371/journal.pone.0049419)
Supplement: Table S2 — Genes characterizing immune response components or chemotaxis that show higher expression in Amela vs Mela tumors but are expressed at lower level in Amela lines in culture than in Amela tumors. (PDF) [file pone.0049419.s006.pdf]

Table S2. Amela tumor-expressed genes characterizing tumor-infiltrating leukocytes.

Gene symbol	Gene name	[b]Log2 ratio Amela/Line	[c]Log2 ratio Amela/Mela	[d]Lineage expression
	[a]*Immune response and chemotaxis*			
Emr1	EGF-like module containing, mucin-like, hormone receptor-like sequence 1	5.94	0.13	M
Ly86	Lymphocyte antigen 86	5.72	1.09	M, L
Mrc1	Mannose receptor, c type 1	5.60	1.12	M
Fcgr3	Fc gamma receptor 3	4.86	0.84*	M
Fbn1	Fibrilin 1	4.77	1.16	S
Fcrls/Msr2	Fc receptor-like S, scavenger receptor	4.60	1.12	M
Ccl12	Chemokine (C-C motif) ligand 12	4.19	1.71	M
Mmp3	Matrix metallopeptidase 3	4.18	2.05	M, S
Cd72	CD72 antigen	4.10	1.85	L, M
C1qa	Complement component 1, q subcomponent, A chain	4.08	1.39	M
Cx3cr1	Chemokine (C-X3-C) receptor 1	3.90	1.51	M, L
Mmp23	Matrix metallopeptidase 23	3.62	1.27	M, S
C1qr1	CD93 antigen	3.35	1.81	M
Csf1r	Colony stimulating factor 1 receptor	2.93	1.10	M
Slamf9	SLAM family member 9	2.86	1.05	M
Ltbp2	Latent transforming growth factor beta binding protein 2	2.78	1.16	S
Ccr1	Chemokine (C-C motif) receptor 1	2.61	2.07	M
Tgfbr2	Transforming growth factor, beta receptor II	2.24	0.96*	L, M
Lsp1	Lymphocyte specific 1	2.19	1.28	L, M
Tlr1	Toll like receptor 1	2.18	1.18	M
Cfh	Complement component factor h	2.01	2.15	M
Ltbp3	Latent transforming growth factor beta binding protein 3	1.57	0.96*	S
Mmp2	Matrix metallopeptidase 2	1.34	0.91*	S

(a) Genes characterizing immune response components or chemotaxis that show higher expression in Amela versus Mela primary tumors but are expressed at lower level in Amela lines in culture than in Amela primary tumors.
(b) Ratio of gene expression as Log2 primary Amela/cultured Amela line > 1 with p value < 0.001;
(c) Ratio of gene expression as Log2 primary Amela/primary Mela > 1 with p value < 0.001;
* Ratio of gene expression as log2 primary Amela/primary Mela between 0 and 1 with p values < 0.05.
(d) Cell lineage with highest expression of the gene taken from the Immunological Genome Project (www.immgen.org) : M for myeloid, L for lymphocytes, S for stromal cells.
