# Supplementary material for: Epithelial-Mesenchymal-Transition-Like and TGFβ Pathways Associated with Autochthonous Inflammatory Melanoma Development in Mice
Source: PLoS One. 2012 Nov 16;7(11):e49419. doi: 10.1371/journal.pone.0049419 (PMC3500287; doi:10.1371/journal.pone.0049419)
Supplement: Table S3 — Table representing the genes highly expressed in Amela tumors having one or multiple conserved Smad binding sites in their promoter (in silico analysis). For each gene, the conserved Smad binding sites (CAGA), their number and their p values are represented. (PDF) [file pone.0049419.s007.pdf]

Gene	nb of sites	1st site	p values	2nd site	p values	3rd site	p value	4th site	p values
Dkk2	4	AGCCAGACAG	4.3e-05	GTCCAGACAT	8.5e-05	TGCCAGACTC	8.5e-05	AGTCAGACAG	8.5e-05
Crispld2	4	ACGCAGACAT	1.9e-05	GGTCAGACAC	4.3e-05	GGCCAGACTG	4.3e-05	AGCTAGACAC	8.5e-05
cnih	3	AGCCAGACAT	4.3e-05	AGTCAGACAT	8.5e-05	AGCTAGACAC	8.5e-05		
Arhgdib	3	ACGCAGACAC	4.5e-06	ATCCAGACAG	5.9e-05	GGTCAGACA	8.5e-05		
CD79a	3	GGCCAGACTG	4.3e-05	GGCCAGACTG	4.3e-05	AGTCAGACTG	8.5e-05		
Gvin1	3	ATCCAGACAC	1.9e-05	TCGTAGACAG	4.3e-05	TGCCAGACTG	5.9e-05		
Pard6a	3	AGCCAGACAC	1.3e-05	ACGTAGACTC	5.9e-05	AGCTAGACAC	8.5e-05		
Uaca	3	AGCCAGACAG	4.3e-05	AGCCAGACAG	4.3e-05	AGGCAGACAC	8.5e-05		
Upp1	3	TGCCAGACTG	5.9e-05	GGGCAGACAC	5.9e-05	ATCCAGACAT	8.5e-05		
Pla2g7	3	AGCTAGACAC	8.5e-05	AGCTAGACAC	8.5e-05	AGTCAGACTG	8.5e-05		
Dpys13	3	GGCCAGACAC	1.3e-05	AGTCAGACAG	8.5e-05	AGTCAGACTG	8.5e-05		
Slc14a1	3	AGTCAGACAC	4.3e-05	ATCCAGACTC	8.5e-05	AGGCAGACAC	8.5e-05		
fhl2	2	ACGTAGACAT	5.9e-05	ACGTAGACAG	5.9e-05				
Il1r1-201	2	TCGCAGACAG	1.3e-05	AGGCAGACAC	8.5e-05				
Stat4	2	ATCCAGACTG	5.9e-05	ATGCAGACAC	8.5e-05				
Ly75	2	ATCCAGACAG	5.9e-05	ATGCAGACAC	8.5e-05				
Ak1	2	GGCCAGACAT	4.3e-05	AGGCAGACAC	8.5e-05				
Mllt11	2	GGCCAGACTG	4.3e-05	GTCCAGACTG	8.5e-05				
Glipr2	2	GGCCAGACAT	4.3e-05	TGCCAGACAG	5.9e-05				
Steap1	2	GGGCAGACAC	5.9e-05	TGCCAGACTC	8.5e-05				
Tbx3	2	GCGTAGACTG	1.9e-05	GGGCAGACAC	5.9e-05				
Spp1	2	AGCCAGACTG	4.3e-05	GTCCAGACAG	8.5e-05				
Pthlh	2	AGGCAGACCG	1.3e-05	AGGTAGACCG	1.9e-05				
Pde3b	2	TGCCAGACAT	5.9e-05	GTCCAGACAG	8.5e-05				
Pvr	2	TGCCAGACAT	5.9e-05	GTCCAGACAT	8.5e-05				
Arnt1	2	AGCCAGACAG	4.3e-05	GTCCAGACAG	8.5e-05				
Gng8	2	ATCCAGACCG	1.6e-06	AGTCAGACAG	8.5e-05				
Sema7a	2	AGCCAGACGG	4.3e-05	GGCCAGACTC	4.3e-05				
Maged1	2	GGGCAGACAC	5.9e-05	AGGCAGACAC	8.5e-05				
Nudt4	2	GCGCAGACAC	4.3e-05	AGCCAGACAT	4.3e-05				
Fkbp10	2	TGCCAGACAG	5.9e-05	AGCTAGACAC	8.5e-05				
Stac2	2	GTCCAGACAG	8.5e-05	AGTCAGACAT	8.5e-05				
Eml1	2	AGGCAGACAC	8.5e-05	CGGCAGACCG	1.9e-05				
Scara3	2	TCGCAGACAC	2.8e-06	AGGCAGACAC	8.5e-05				
Kdelr3	2	CGCCAGACAC	5.9e-05	GGTCAGACAG	8.5e-05				
Cdh6	2	ATCCAGACAG	5.9e-05	ATGCAGACAC	8.5e-05				
Has2	2	ACGCAGACAG	1.3e-05	TGCCAGACAC	1.9e-05				
Dab2	2	AGCCAGACAC	1.3e-05	GTCCAGACAC	4.3e-05				
Vegfa	2	ACGCAGACTC	1.9e-05	ATCCAGACAG	5.9e-05				
Msln	2	AGCCAGACAC	1.3e-05	TGCCAGACAC	1.9e-05				
Stambpl1	2	AGCCAGACAC	1.3e-05	AGGTAGACCG	1.9e-05				
Gprc5b	1	GGTCAGACCG	2.8e-06						
Rgs2	1	TGCCAGACAT	5.9e-05						
Rgs16	1	GTCCAGACGG	8.5e-05						
Htr5b	1	GGTCAGACGG	8.5e-05						
Il1r1-202	1	GTCCAGACAG	8.5e-05						
hoxd8	1	TGCCAGACAC	1.9e-05						
Snail	1	GGCCAGACTC	4.3e-05						
Dnm1	1	GTCCAGACAG	8.5e-05						
Tfpi	1	GGCCAGACAC	1.3e-05						
Zfhx4	1	AGGCAGACAC	8.5e-05						
Pdgfc	1	AGTCAGACAG	8.5e-05						
Cyr61	1	GGCCAGACAC	1.3e-05						
Hs2st1	1	CGGCAGACCG	1.9e-05						
Sec24d	1	AGCCAGACAC	1.3e-05						
Gpr149	1	GGCCAGACTG	4.3e-05						
Traml11	1	ATCCAGACAG	5.9e-05						
Cp	1	AGTCAGACA	4.3e-05						
Prkacb	1	AGTCAGACAG	8.5e-05						
Epha7	1	ATCCAGACAC	1.9e-05						
Mxra8	1	AGGCAGACAC	8.5e-05						
Tusc1	1	ATCCAGACAT	8.5e-05						
Kpna6	1	AGTCAGACA	4.3e-05						
Trit1	1	AGCTAGACCG	1.3e-05						
Steap2	1	GGTCAGACTG	8.5e-05						
IL6	1	TGCCAGACAG	5.9e-05						
Usp18	1	GCGTAGACTC	1.9e-05						
Camk1	1	AGCTAGACAC	8.5e-05						
Cald1	1	AGGCAGACAC	8.5e-05						
Axl	1	AGCTAGACAC	8.5e-05						
Nupr1	1	GGCCAGACAT	4.3e-05						
Tead2	1	ATCCAGACAT	8.5e-05						
Il11	1	GGCCAGACAG	4.3e-05						
Cdyl2	1	AGCCAGACAT	4.3e-05						
Rasgrf1	1	GCGCAGACCG	2.8e-06						
Itm2a	1	GTCCAGACAG	8.5e-05						
Sh3kbp1	1	GTCCAGACAG	8.5e-05						
Flna	1	AGTCAGACTG	8.5e-05						
Cnn2	1	GCGCAGACAG	8.5e-05						
Aldh112	1	TGCCAGACAG	5.9e-05						
CCl2	1	AGCCAGACAT	4.3e-05						
Mmd	1	GCGTAGACAT	1.9e-05						
Ccl7	1	AGCCAGACAC	1.3e-05						
Ccl5	1	TGCCAGACAG	5.9e-05						
hoxb7	1	ACGCAGACTG	1.3e-05						
Slc16a3	1	GTCCAGACTG	8.5e-05						
Ntn1	1	GTCCAGACTG	8.5e-05						
Lamb1-1	1	AGCCAGACAG	4.3e-05						
Loxl2	1	ATGCAGACAC	8.5e-05						
Bmp1	1	GGTCAGACTG	8.5e-05						
Ghr	1	GCGTAGACAG	1.3e-05						
Rail4	1	AGGCAGACAC	8.5e-05						
Ghr	1	GCGTAGACAG	1.3e-05						
Adamts1	1	AGCCAGACAG	4.3e-05						
Cdh2	1	TGCCAGACAC	1.9e-05						
Dpys13	1	GGTCAGACTG	8.5e-05						
Pcsk5	1	GGCCAGACAC	1.3e-05						
Insl6	1	GGCCAGACAG	4.3e-05						
Pik3ap1	1	ATGCAGACAC	8.5e-05						

Table S3
