# Supplementary material for: Epithelial-Mesenchymal-Transition-Like and TGFβ Pathways Associated with Autochthonous Inflammatory Melanoma Development in Mice
Source: PLoS One. 2012 Nov 16;7(11):e49419. doi: 10.1371/journal.pone.0049419 (PMC3500287; doi:10.1371/journal.pone.0049419)
Supplement: Figure S1 — Unsupervised Hierarchical Clustering of melanoma tumors and healthy skin samples. Each row represents a gene, and each column represents a sample. Each experimental sample is represented by 2 or 3 values associated to 2 or 3 different hybridizations. The expression level of each gene in a single tumor is relative to its median abundance across all tumors and is depicted according to a color scale in which red and green expression levels are, respectively, above and below the median. The magnitude of deviation from the median is represented by the color saturation. The dendrogram of samples (matrix on top) represents overall similarities in gene expression profiles. Four clusters are shown: - clusters of genes highly expressed selectively in Amela (A) or in Mela (B) tumors; - clusters of genes which expression is up-regulated (C) or down-regulated (D) in both tumors as compared to healthy skin. (PDF) [file pone.0049419.s001.pdf]

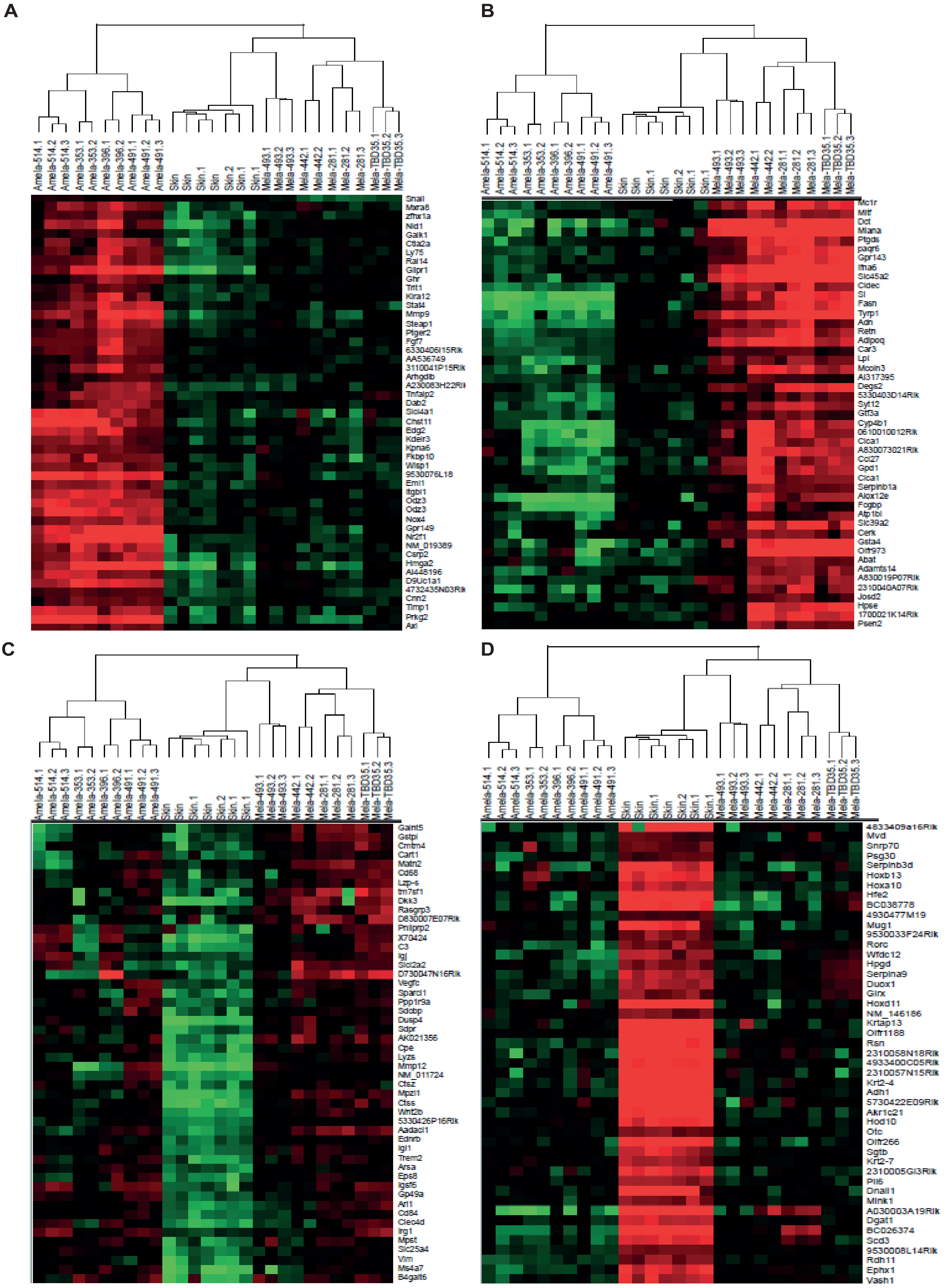
A
Amela-514.1
Amela-514.2
Amela-514.3
Amela-353.1
Amela-353.2
Amela-396.1
Amela-396.2
Amela-491.1
Amela-491.2
Amela-491.3
Skin
Skin
Skin.1
Skin
Skin.2
Skin.1
Skin.1
Mela-493.1
Mela-493.2
Mela-493.3
Mela-442.1
Mela-442.2
Mela-281.1
Mela-281.2
Mela-281.3
Mela-TBD35.1
Mela-TBD35.2
Mela-TBD35.3
Snail
Mxra8
zfhx1a
Nid1
Galk1
Ctla2a
Ly75
Ral14
Gilpr1
Ghr
Trit1
Klra12
Stat4
Mmp9
Steap1
Ptger2
Fgf7
6330406I15Rik
AA536749
3110041P15Rik
Arhgdib
A230083H22Rik
Tnfaip2
Dab2
Slc4a1
Chst11
Edg2
Kdelr3
Kpna6
Fkbp10
Wisp1
9530076L18
Emil1
Itgbl1
Odz3
Odz3
Nox4
Gpr149
Nr2f1
NM_019389
Csrp2
Hmga2
AI448196
D9Ucla1
4732435N03Rik
Cnn2
Timp1
Prkg2
Axl
B
Amela-514.1
Amela-514.2
Amela-514.3
Amela-353.1
Amela-353.2
Amela-396.1
Amela-396.2
Amela-491.1
Amela-491.2
Amela-491.3
Skin
Skin
Skin.1
Skin
Skin.2
Skin.1
Skin.1
Mela-493.1
Mela-493.2
Mela-493.3
Mela-442.1
Mela-442.2
Mela-281.1
Mela-281.2
Mela-281.3
Mela-TBD35.1
Mela-TBD35.2
Mela-TBD35.3
Mc1r
Mitf
Dct
Mlana
Ptgds
paqr6
Gpr143
Itna6
Slc45a2
Cidec
Sl
Fasn
Tyrp1
Adn
Retn
Adipoq
Car3
Lpl
Mcoln3
AI317395
Degs2
5330403D14Rik
Syt12
Gtf3a
Cyp4b1
0610010012Rik
Clca1
A830073021Rik
Ccl27
Gpd1
Clca1
Serpinb1a
Alox12e
Fcgbp
Atp1bl
Slc39a2
Cerk
Gsta4
Olfr973
Abat
Adamts14
A830019P07Rik
2310040A07Rik
Josd2
Hpse
1700021K14Rik
Psen2
C
Amela-514.1
Amela-514.2
Amela-514.3
Amela-353.1
Amela-353.2
Amela-396.1
Amela-396.2
Amela-491.1
Amela-491.2
Amela-491.3
Skin
Skin
Skin.1
Skin
Skin.2
Skin.1
Skin.1
Mela-493.1
Mela-493.2
Mela-493.3
Mela-442.1
Mela-442.2
Mela-281.1
Mela-281.2
Mela-281.3
Mela-TBD35.1
Mela-TBD35.2
Mela-TBD35.3
Galnt5
Gstpi
Cmtm4
Cart1
Matn2
Cd68
Lzp-s
Im7sf1
Dkk3
Rasgrp3
D830007E07Rik
Pnliprp2
X70424
C3
Igj
Slc2a2
D730047N16Rik
Vegfc
Sparcl1
Ppp1r9a
Sdcbp
Dusp4
Sdpr
AK021356
Cpe
Lyzs
Mmp12
NM_011724
Ctsz
Mpzl1
Ctss
Wnt2b
5330426P16Rik
Aadacl1
Ednrb
Igl1
Trem2
Arsa
Eps8
Igsf6
Gp49a
Arl1
Cd84
Clec4d
Irg1
Mpst
Slc25a4
Vim
Ms4a7
B4galt6
D
Amela-514.1
Amela-514.2
Amela-514.3
Amela-353.1
Amela-353.2
Amela-396.1
Amela-396.2
Amela-491.1
Amela-491.2
Amela-491.3
Skin
Skin
Skin.1
Skin
Skin.2
Skin.1
Skin.1
Mela-493.1
Mela-493.2
Mela-493.3
Mela-442.1
Mela-442.2
Mela-281.1
Mela-281.2
Mela-281.3
Mela-TBD35.1
Mela-TBD35.2
Mela-TBD35.3
4833409a16Rik
Mvd
Snrp70
Psg30
Serpinb3d
Hoxb13
Hoxa10
Hfe2
BC036778
4930477M19
Mug1
9530033F24Rik
Rorc
Wfdc12
Hpgd
Serpina9
Duox1
Glrx
Hoxd11
NM_146166
Krtap13
Olfr1188
Rsn
2310058N18Rik
4933400C05Rik
2310057N15Rik
Krt2-4
Adh1
5730422E09Rik
Akr1c21
Hod10
Otc
Olfr266
Sgtb
Krt2-7
2310005G13Rik
Pli6
Dnali1
Mink1
A030003A19Rik
Dgat1
BC026374
Scd3
9530008L14Rik
Rdh11
Ephx1
Vash1

Figure S1
